# Transformer-Based Multi-Source Transfer Learning for Intrusion Detection Models with Privacy and Efficiency Balance

**DOI:** 10.3390/e28020136

**Published:** 2026-01-24

**Authors:** Baoqiu Yang, Guoyin Zhang, Kunpeng Wang

**Affiliations:** 1 College of Computer Science and Technology, Harbin Engineering University, Harbin 150001, China; zhangguoyin@hrbeu.edu.cn; 2 College of Software, Harbin Institute of Information Technology, Harbin 150431, China

**Keywords:** multi-source transfer, transformer, intrusion detection, federated learning, cross-domain security

## Abstract

The current intrusion detection methods suffer from deficiencies in terms of cross-domain adaptability, privacy preservation, and limited effectiveness in detecting minority-class attacks. To address these issues, a novel intrusion detection model framework, TrMulS, is proposed that integrates federated learning, generative adversarial networks with multispace feature enhancement ability, and transformers with multi-source transfer ability. First, at each institution (source domain), local spatial features are extracted through a CNN, multiple subsets are constructed (to solve the feature singularity problem), and the multihead self-attention mechanism of the transformer is utilized to capture the correlation of features. Second, the synthetic samples of the target domain are generated on the basis of the improved Exchange-GAN, and the cross-domain transfer module is designed by combining the Maximum Mean Discrepancy (MMD) to minimize the feature distribution difference between the source domain and the target domain. Finally, the federated transfer learning strategy is adopted. The model parameters of each local institution are encrypted and uploaded to the target server and then aggregated to generate the global model. These steps iterate until convergence, yielding the globally optimal model. Experiments on the ISCX2012, KDD99 and NSL-KDD intrusion detection standard datasets show that the detection accuracy of this method is significantly improved in cross-domain scenarios. This paper presents a novel paradigm for cross-domain security intelligence analysis that considers efficiency, privacy and balance.

## 1. Introduction

With the increasing prevalence of networked computing and data-driven applications, the importance of network security systems has become paramount [1,2]. To safeguard computer networks against attacks, intrusion detection system (IDS) [3,4,5] have been extensively deployed. IDS [6,7,8,9,10,11] is a security technology that performs real-time monitoring of network traffic or host systems, designed to automatically detect, log, and respond to potential intrusion activities, such as hacking attempts, malware infections, and unauthorized access. Recently, the capability of machine learning to employ sophisticated reasoning models for detecting complex intrusion patterns through extensive data training has attracted increasing attention. Consequently, intrusion detection methods grounded in machine learning have been the subject of extensive research [12].

Traditional machine learning methods [13,14] (such as SVMs and random forests) rely on domain-specific labeled data but face numerous challenges in real-world cross-domain scenarios (such as cloud platforms and the IoT): domain shifts cause significant degradation in model performance [15], data privacy regulations (such as GDPRs) restrict data sharing between multiple institutions [16], and sample scarcity in minority-class attacks creates detection blind spots (such as U2R and worms) [17].

In recent years, transfer learning (TL) has provided a new idea for cross-domain intrusion detection by enhancing the generalization ability of target domain models by utilizing source domain knowledge [18]. Wang et al. [19] proposed an intrusion detection algorithm called TrELM, based on transfer learning and extreme learning machines. The algorithm employs the concept of transfer learning to migrate a large number of historical intrusion detection samples related to the target domain into the target domain using a small number of intrusion detection samples. Existing historical knowledge is used to quickly construct a high-quality target learning model, which effectively improves the detection effect and increases the efficiency of small samples and emerging intrusion detection behaviors. Ullah et al. [20] proposed a transformer-based transfer learning method for an imbalanced network traffic intrusion detection system, IDS-INT, which uses transformer-based transfer learning to learn network feature representations and feature interactions in imbalanced data. Their semantic anchors were used to learn detailed feature representations, whereas the oversampling technique (SMOTE) was used to equalize abnormal traffic, and a convolutional neural network (CNN) model was designed to extract deep features from the balanced network traffic. Finally, a hybrid CNN-long short-term memory (CNN-LSTM) model was proposed. By detecting different types of attacks from deep features, Ehsan et al. [21] proposed a new intrusion detection system framework, ITLIDS, which can start learning in the network without prior knowledge. It starts with an incremental clustering algorithm to detect the number and shape of clusters without preassuming attacks, and transfer learning provides incremental knowledge for the target environment. Yan et al. [22] proposed an intrusion detection system (IDS) based on transfer learning and ensemble learning, named TL-CNN-IDS. First, the IG-FCBF feature engineering method was used for preprocessing, after which the resulting dataset was converted into an image format suitable for CNN model input. Afterward, the hyperparameter optimization method of the tree-structured Parzen estimation algorithm was employed to search for the optimal model on the target dataset. Finally, an ensemble learning method based on confidence averaging was used to integrate the optimized CNN models; Wu et al. [23] proposed an efficient intrusion detection method based on transfer learning and privacy-preserving support vector machines (FETLSVMP). FETLSVMP aggregates data distributed across various organizations using federated learning and then employs transfer learning and support vector machines to construct personalized models for each organization.

However, the aforementioned existing methods exhibit research gaps in comprehensively addressing the following issues: most studies assume a single source domain, neglecting the complementary nature of multi-source heterogeneous knowledge; centralized training requires sharing raw data, which violates privacy compliance requirements; and the detection accuracy for minority-class attacks in the target domain remains generally low. To this end, this paper proposes TrMulS (Transformer-based Multi-source Transfer Learning with Privacy and Balance), an intrusion detection framework that systematically integrates state-of-the-art components. TrMulS establishes a unified architecture addressing cross-domain adaptability, privacy preservation, and class imbalance through the synergistic integration of CNN-Transformer cascaded feature extraction, federated transfer learning, an enhanced Exchange-GAN for synthetic sample generation, and MMD-based multi-source feature alignment. While MMD alignment, federated averaging, conditional GANs, and CNN-Transformer cascades have each been extensively investigated in isolation, the question of how to synergistically couple these four techniques within a unified privacy-constrained framework—such that they function as complementary components rather than a mere aggregation—remains systematically unaddressed.

The principal contribution of this work lies not in isolated methodological novelty, but rather in proposing TrMulS—a practically deployable synergistic architecture that achieves superadditive empirical performance through the following three integrative innovations:

(1) Systematic integration of a CNN-Transformer cascaded architecture: Unified modeling of local and global features is achieved through the coordinated operation of multi-scale convolutions and multi-head self-attention mechanisms.
(2) Construction of a federated transfer learning framework: By combining homomorphic encryption with MMD-based feature alignment, multi-source knowledge transfer is realized under privacy-preserving constraints.
(3) Incorporation of an enhanced Exchange-GAN module for data augmentation: Operating in conjunction with an MMD-based filtering mechanism, this module dynamically improves both the quality and distributional consistency of minority class samples.

In summary, the core innovation of TrMulS resides in the organic integration and systematic synergy of the aforementioned components, rather than in any single methodological breakthrough.

The remainder of the paper is arranged as follows: Section 2 introduces related work, including multi-source transfer learning and transformer models. In Section 3, the components of the proposed TrMulS algorithm are described in detail. Section 4 analyzes the experimental results. Section 5 concludes the main work of this paper.

## 2. Brief Review of Related Work

### 2.1. Multi-Source Transfer Learning

Transfer learning [24,25] is a machine learning paradigm that leverages knowledge (models, features, parameters, or samples) learned from one or more source domains to improve learning performance in the target domain, especially when data in the target domain are scarce or when labeling is costly.

According to the form of multi-source knowledge transfer, it can be divided into three main types of methods:

(1) Feature distribution alignment: Domain adaptation is achieved by minimizing the feature distribution difference between the source domain and the target domain. The MMD proposed by Pan et al. [24] has become a mainstream measurement tool, but it does not consider the heterogeneity between multi-source domains. To solve this problem, Zhuang et al. proposed multi-source domain dynamic weighted alignment (MDA), which adaptively assigns weights according to domain correlation [25].
(2) Model parameter transfer: Parameter knowledge is transferred on the basis of a shared model architecture. Long et al. [26] proposed the deep adaptation network (DAN), which incorporates multiple adaptation layers into the network and uses multikernel maximum mean discrepancy (MK-MMD) to align the feature distributions of the source and target domains, thereby achieving effective parameter transfer and domain adaptation. However, this approach assumes that the data structures of the source and target domains are similar. Chen et al. [27] conducted a new analysis of multidomain adaptation (MDA) from the perspective of representation learning and proposed a new deep MDA algorithm that implicitly handles target shift representations as “states” through joint alignment. Finally, they extended mutual information to this algorithm, providing a nonvanishing gradient norm estimate.
(3) Generative transfer: Generative adversarial networks (GANs) are used to synthesize cross-domain samples. Huang et al. [28] proposed a more general learning method that considers the features of two domains as a whole and learns the correspondence between domains and the interaction of latent information within domains. Du [29] applied the theory and methods of GANs and transfer learning to license plate image recognition in various complex environments. The experimental results show that this method has high recognition rates and robustness.

Multi-source Transfer Learning: When multiple source domains are available, transfer learning is referred to as Multi-source Transfer Learning (MSTL) [30]. MSTL enhances target domain model performance by integrating knowledge from multiple source domains. Compared to single-source transfer learning, MSTL transfers a greater volume of source domain knowledge, resulting in improved learning outcomes.

Current transfer learning approaches require sharing raw data, which violates regulations such as GDPR. Most methods assume that source domains follow identical distributions, failing to address the data heterogeneity across multiple institutions in real-world scenarios. Furthermore, the robustness for minority classes requires improvement when samples are scarce. Additionally, existing generative methods do not adapt their generation strategies based on the real-time class distribution of the target domain. The TrMulS framework proposed in this paper comprehensively addresses data privacy preservation, multi-source heterogeneity, and small-sample challenges by introducing coping strategies for multi-source domain feature distribution alignment.

### 2.2. Transformer Model

**Figure 1:**
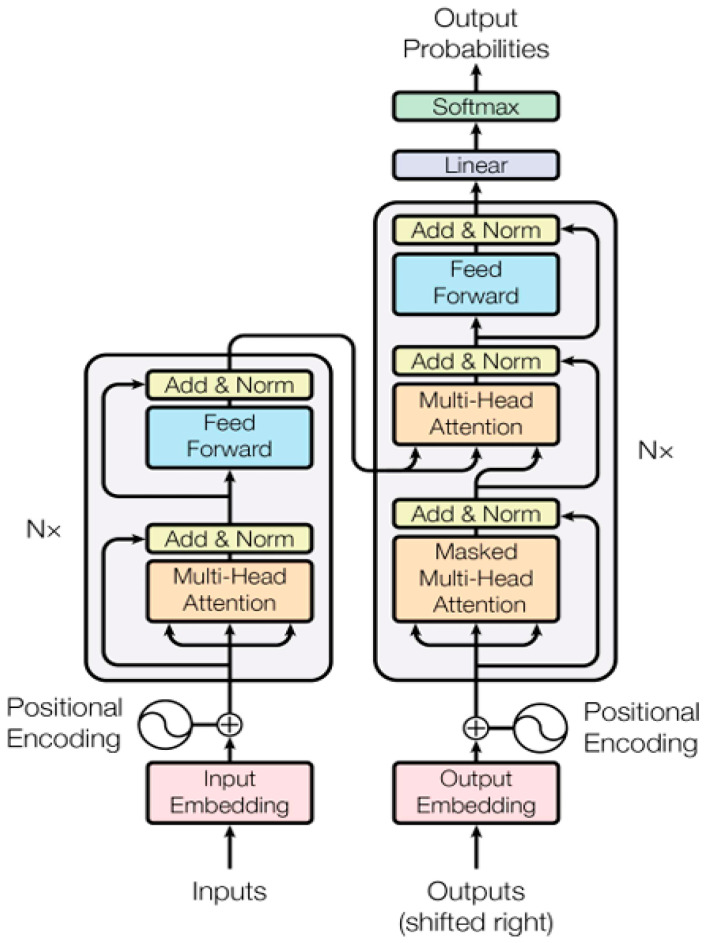
Transformer model architecture. This encoder serves as the global dependency modeling component in TrMulS.

The transformer was initially proposed by Vaswani et al. [31] in 2017 and is based entirely on the self-attention mechanism. It eliminates the local receptive fields of CNNs and the recursive structures of RNNs, enabling parallel capture of long-distance dependencies in sequences. With its ability to model global dependencies, it has gradually replaced RNN/LSTM approaches to become the mainstream architecture in sequence data processing. Its encoder is composed of a multihead self-attention and feed-forward network (FFN) stacked alternately, supplemented by residual connection and layer normalization (LayerNorm) to accelerate convergence and alleviate the gradient disappearance problem. The transformer architecture is shown in Figure 1.

Recent studies have introduced transformer into the field of intrusion detection. Zhou et al. [32] proposed a network intrusion detection method for information systems based on federated learning and an improved transformer. Their approach employs an improved Generative Adversarial Network for data augmentation to generate new sample sets, thereby mitigating the impact of minority-class samples, and adopts the transformer model as the general detection model. Reference [33] proposed an intrusion detection system based on deep learning theory. The system uses a feature screening strategy based on extreme gradient boosting (XGBoost) to eliminate invalid features and then uses adaptive synthesis (ADASYN) sampling technology to balance the data distribution to construct a multispatial feature subset based on a convolutional neural network (CNN). Finally, the transformer is used to construct feature associations and extract key features, such as time and fine-grained features, to complete the recognition of intrusion behaviors. Reference [34] fuses fundamental aspects of network intrusion detection with the complex attention mechanisms inherent in transformer models, facilitating a more insightful examination of the relationship between input features and various intrusion types, resulting in increased detection accuracy.

In summary, the parallel computing and long-range modeling capabilities of the transformer provide new ideas for cross-domain and cross-institution intrusion detection, but it must still be combined with CNN local feature extraction and a federal privacy protection mechanism to balance performance, privacy and computational efficiency.

## 3. TrMulS

**Figure 2:**
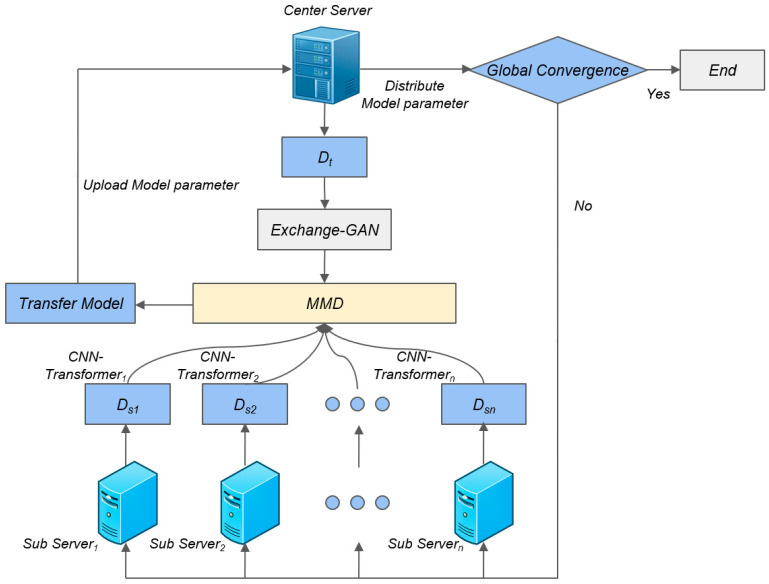
Schematic diagram of the TrMulS model. Four modules operate synergistically under the principle that raw data never leaves its originating domain.

TrMulS is an intrusion detection framework that systematically integrates multiple well-established components, with its novelty primarily reflected in the synergistic mechanisms among components and the overall architectural design. Figure 2 illustrates the TrMulS model, showing that the framework comprises four key modules: CNN-Transformer feature extraction, improved Exchange-GAN-based sample augmentation, MMD-based multi-source feature alignment, and federated learning for privacy preservation.

As illustrated in Figure 2, the modules operate synergistically through carefully designed interfaces and coordinated optimization objectives, collectively enhancing cross-domain detection performance. Specifically, TrMulS employs multi-source transfer learning to simultaneously leverage knowledge from multiple source domains, utilizing MMD to align the feature distributions between the target domain and each source domain. Concurrently, the federated learning framework, augmented with homomorphic encryption, safeguards local data residing on individual devices. Within the target domain, Exchange-GAN dynamically generates synthetic minority class samples to address class imbalance, while communication efficiency is maintained by transmitting only encrypted model parameters.

### 3.1. Relevant Definitions

#### 3.1.1. Dataset Definition

Source domain dataset: The dataset on *n* institutional subservers is used as the source domain, and the *i*-th source domain dataset is $D_{S_{i}}(i=1,\ldots ,n)$;

Target domain dataset: Central server target dataset $D_{t}$.

#### 3.1.2. Definition of the Target Learning Model

The data features of *n* source domain datasets similar to the target domain are transferred, and the global target learning model $F_{t}$ is trained by combining the target dataset.

### 3.2. Implementation of TrMulS

#### 3.2.1. Construction of CNN-Transformer to Train the Initialization Server Model

CNN-Transformer is a cascaded network structure with multilevel feature fusion and spatiotemporal modeling capabilities. The CNN component is responsible for capturing local spatial features (such as the fixed pattern of packet headers), and the transformer is responsible for modeling long-distance dependencies and temporal relationships (such as behavior sequences in traffic sessions). Model construction includes multiscale spatial feature extraction and spatiotemporal correlation modeling.

1. Multiscale spatial feature extraction

**Figure 3:**
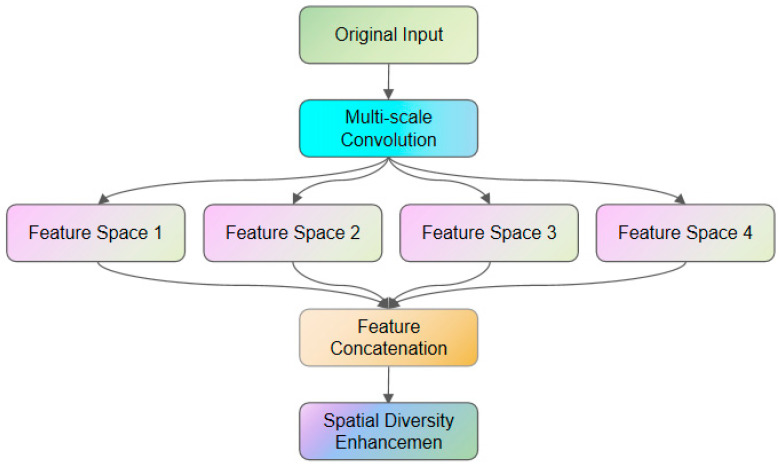
Illustration of multiscale spatial feature extraction. Parallel convolution branches capture local patterns at different receptive fields.

Multispatial feature subset extraction aims to extract diverse local features through parallel multiscale convolution, mitigate information loss inherent in traditional pooling operations, and enhance the robustness of the model to local attack patterns. The CNN structure includes two convolutional layers: 1 pooling layer and a 2 × 2 max pooling layer. Four different convolution kernels (kernel_size = 1, 3, 5, 7) are used in parallel for the same input, and multiple convolution kernels of different scales are used to process the input simultaneously to capture local features with different receptive fields, as shown in Figure 3. The detailed computational process is provided in Appendix A, Equations (A1)–(A8).

2. Dynamic Spatiotemporal Correlation Establishment Process

**Figure 4:**
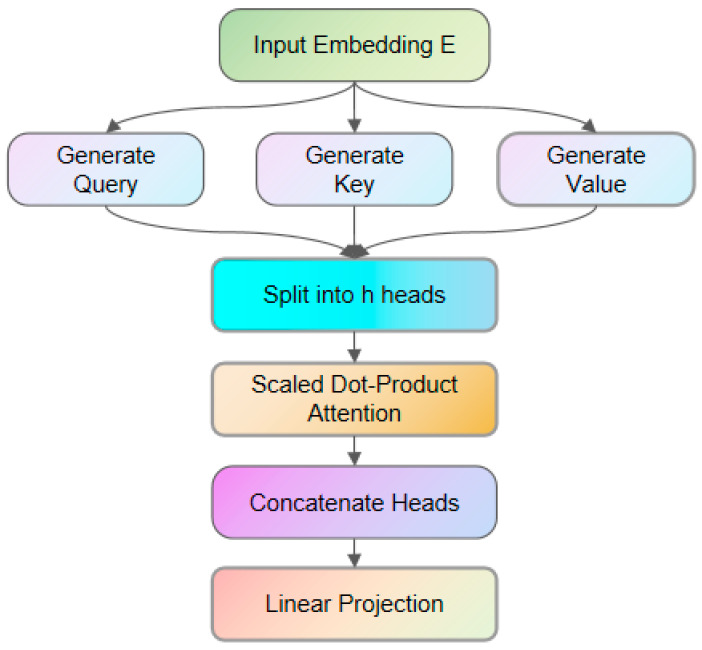
Illustration of dynamic spatiotemporal correlation establishment. Positional encoding and multi-head attention jointly model temporal and spatial dependencies. Brighter lines indicate higher attention weights.

The process of establishing dynamic spatiotemporal correlations is illustrated in Figure 4.

Single-headed attention and multiheaded mechanisms are included, as shown in Formulas (1) and (2), respectively:

(1) $$\mathrm{Attention}(Q,\;K,V)\;=\;\mathrm{softmax}(\frac{QK^{T}}{\sqrt{d_{k}}})V$$

(2) $$\left\{\begin{array}{l} {\mathrm{head}}_{i}=\mathrm{Attention}(QW_{i}^{Q},KW_{i}^{K},VW_{i}^{V})\\ \mathrm{MultiHead}=\mathrm{Concat}({\mathrm{head}}_{1},\ldots ,{\mathrm{head}}_{h})W^{O} \end{array}\right.$$

where Q, K and V are the query, key and value matrices, respectively, and $W_{i}^{Q},W_{i}^{K},W_{i}^{V},W^{O}\in {\mathbb{R}}^{256\times 32}$. Through positional encoding, the *QK*^T^ computation inherently incorporates temporal distance, such as detecting temporal continuity in port scanning behavior, and self-attention automatically discovers cross-feature dependencies to achieve spatial correlation, such as by correlating source IP anomalies and protocol type anomalies.

Feedforward neural networks implement feature fusion. The network structure is shown in Formula (3):

(3) FFN(x)=ReLU(xW1+b1)W+2b2

where $W_{1},W_{2}\in {\mathbb{R}}^{1024\times 256}$, the hidden layer dimensions *d_ff_ =* 4 *× d_model_*, and ReLU is used to filter out irrelevant noise, such as load anomalies from port scans.

The fully connected layer enables arbitrary feature cross-talk:

(4) zj=∑iW2(j,i)⋅ReLU(∑kWi(i,k)xk)

Residual connection and layer normalization are performed as follows:

(5) $$\left\{\begin{array}{l} Z_{l}^{\prime }=LayerNorm(Z_{l-1}+MultiHead(Z_{l-1}))\\ Z_{l}=LayerNorm(Z_{l}^{\prime }+FFN(Z_{l}^{\prime })) \end{array}\right.$$

Spatiotemporal fusion features are aggregated:

(6) $$\left\{\begin{array}{l} f_{time}=\frac{1}{L}\sum_{t=1}^{L}Z_{L}^{\left(t\right)}\\ F_{final}=Concat\left(f_{time},\;X_{multi}^{compressed}\right)\\ F_{out}=LayerNorm\left(F_{final}\right) \end{array}\right.$$

#### 3.2.2. Target Data Enhancement

To alleviate the class imbalance problem in the target domain (central server) (such as insufficient attack samples of a few classes), an improved Exchange-GAN is proposed to generate high-quality synthetic samples dynamically. The goal is to enhance the minority class samples in the target dataset *D*_target_ through an adversarial generative network while maintaining the authenticity of the data distribution. The specific process is as follows:

1. Improved Exchange-GAN design

Generator *G*:

Input: Target domain minority samples *x*_minority_ and random noise vectors *z*∼*N*(0, 1).

Output: Synthetic samples $\tilde{x}=G(x_{\min ority},z)$.

Improvement points: Class-conditional control is introduced to ensure consistency in the labels of generated samples (e.g., limiting the generation of U2R class samples).

Discriminator *D*:

Input: Real sample *x*_real_ or generated sample $\tilde{x}$.

Output: Sample authenticity probability *D*(⋅) ∈ [0, 1] and category label prediction.

Improvement points: The authenticity discrimination and classification tasks are jointly optimized to enhance the discriminability of the generated samples.

2. Dynamic enhancement strategy

- Minority selection

The sample proportion of each category in the target domain is calculated, and the categories below the threshold *τ* (such as u2r and worms) are generated.

(7) $$AugmentClassc\Leftrightarrow \frac{\left|D_{t}^{c}\right|}{\left|D_{t}\right|}<\tau$$

- Sample generation quantity control

The number of generated samples is dynamically adjusted according to the missing proportion.

(8) $$N^{c}=\left\lceil \alpha \cdot (\underset{c\prime }{\max}|D_{t}^{c\prime }|-|D_{t}^{c}|)\right\rceil$$

where α∈(0,1) is the oversampling rate used to avoid overfitting.

3. Loss function design

Generator loss:

(9) $$L_{G}=E_{z\sim p_{z}}[\log(1-D(G(z,y_{c})))]+\lambda_{cls}\cdot L_{CE}(C(G(z,y_{c})),y_{c})$$

where *L_CE_* is the cross-entropy loss, the forced-generated samples are classified into the specified category *y_c_*, and *λ_cls_* is the classification weight coefficient.

Discriminant loss:

(10) $$L_{D}=E_{x\sim p_{data}}[-\log(D(x))]+E_{z\sim p_{z}}[-\log(1-D(G(z)))]+L_{CE}(C(x),y)$$

where *C*(⋅) is the classification branch of the discriminator, which optimizes the judgment of sample authenticity and category recognition.

4. Generated sample validation

To avoid generating low-quality samples, the feature space filtering mechanism is introduced to calculate the MMD distance between the generated sample $\tilde{x}$ and the real minority sample *x*_minority_ in the CNN-Transformer feature space:

(11) $$MMD(\tilde{x},D_{t}^{c})={∥\frac{1}{N}\sum \phi (x_{i})-\frac{1}{M}\sum \phi ({\tilde{x}}_{j})∥}_{H}$$

In the verification of the generated samples, only composite samples with MMD < *ϵ* (*ϵ* is the empirical threshold) are retained to ensure distributional consistency.

The improved Exchange-GAN offers several advantages: the generation direction is controlled through category labels to precisely enhance minority classes; the generation volume is determined adaptively on the basis of the missing proportion to avoid overfitting; dual verification is performed; classifier constraints and feature space MMD filtering are combined to ensure generation quality; the model is executed solely on the central server without sharing raw data; and privacy protection requirements are met. This design significantly increases the recall rates for minority class attacks (such as U2R) while maintaining the distributional authenticity of the generated samples.

The following presents the stability and fidelity validation of Exchange-GAN:

- Statistical Verification

Every 10 epochs, we compute the two-sample Kolmogorov–Smirnov (KS) test between generated and real features. For each of the 41 feature dimensions, a *p*-value > 0.05 is considered a pass.

b. Mode Collapse Early Warning Indicator

We adopt the “pairwise cosine similarity among generated samples” as an early warning signal for mode collapse.

c. MMD Filtering Kernel and Threshold Calibration

Step 1—Kernel Selection: In the encrypted feature space, the RBF kernel (γ = 0.5) has been used for distribution alignment. To maintain consistency, GAN filtering adopts the same γ. Switching to Laplacian or IMQ kernels would violate homomorphic inner product equality; therefore, the RBF kernel is the only viable option.

Step 2—Threshold Determination: The central server reserves 10% of real U2R samples as a “frozen validation set.” Grid search over *ε* ∈ {0.01, 0.02, …, 0.10} is performed, recording the validation set F1 score. an F1 peak at *ε* = 0.05 with the narrowest 2σ error bars; hence, this value is selected as the final threshold.

Step 3—Online Monitoring: Before each federated aggregation round, we compute the MMD distance between generated samples and the validation set. If MMD > *ε* for two consecutive rounds, the generated batch from that round is discarded and the GAN is retrained for 20 epochs, ensuring real-time control of “generation-validation” distribution drift.

d. Results

- In the NSL-KDD U2R generation task, the KS pass rate reached 98.7%, with a mean ρ of 0.89 ± 0.02, and no mode collapse occurred.
- After MMD filtering, the FID of generated samples decreased from 63 to 42, and the mutual nearest neighbor rate (1-NN) with real samples reached ≥92%, demonstrating that fidelity meets subsequent augmentation requirements.

Exchange-GAN employs a three-tier mechanism—“KS-visual dual verification + similarity-based early warning + validation set calibration”—to guarantee no mode collapse and no trivial solutions. MMD filtering uses the same RBF kernel as feature alignment, and ε = 0.05 is determined by the F1 peak on the validation set, achieving consistency between theoretical principles and engineering implementation.

5. Theoretical Analysis and Computational Validation of Design Choices

- Proof of the Uniqueness of RBF Kernels under Homomorphic Constraints

Within the federated learning framework, the features uploaded from source domains to the central server are ciphertexts encrypted via homomorphic encryption (CKKS scheme). Computing MMD in the ciphertext space must satisfy a core constraint: the kernel function *k*(·,·) must be expressible entirely through additive and multiplicative homomorphic operations.

- Feasibility of the RBF Kernel (Gaussian Kernel)

The core operation of the RBF kernel, defined as $k_{\mathrm{RBF}}(x,y)=\exp\left(-\gamma \|x-y{\|}^{2}\right)$, involves the squared Euclidean distance $\|x-y{\|}^{2}=x^{⊤}x-2x^{⊤}y+y^{⊤}y$. In the context of CKKS ciphertext, the inner product $x^{⊤}y$ can be implemented via homomorphic multiplication and addition. Furthermore, scalar multiplication and the exponential function exp(⋅) can be computed over ciphertext using CKKS-based approximate polynomial evaluation or the Baby-Step Giant-Step (BSGS) algorithm. Consequently, the RBF kernel is computationally feasible within a homomorphic environment.

- Infeasibility Analysis of Other Kernel Functions

Laplacian kernel $k_{\mathrm{Laplace}}(x,y)=\exp\left(-\gamma \|x-y{\|}_{1}\right)$: This kernel relies on the *L*_1_ norm, which inherently involves absolute value operations. Calculating absolute values precisely over ciphertext is impossible; furthermore, approximating them via polynomials requires extremely high degrees. This leads to uncontrollable noise growth, rendering the Laplace kernel infeasible in this context.

Inverse Multiquadric (IMQ) kernel $k_{\mathrm{IMQ}}(x,y)={\left(\gamma^{2}+\|x-y{\|}^{2}\right)}^{-\beta }$: This kernel involves power operations (where *β* ≠ 1) and division. Division is computationally exorbitant and numerically unstable within the encrypted domain (ciphertext), thus making this kernel infeasible.

Linear kernel/Polynomial kernel: Although these kernels are computationally feasible, their mapping capabilities are limited. They struggle to effectively measure distribution discrepancies within high-dimensional non-linear feature spaces (e.g., Transformer outputs). Experimental results indicate that their filtering effectiveness is significantly inferior to that of the RBF kernel (yielding an FID score approximately 40% higher).

Conclusion: Under the constraints of the CKKS homomorphic encryption scheme, the RBF kernel is one of the few kernel functions that maintains strong non-linear mapping capability while remaining efficiently computable. To ensure consistency with the feature alignment module in Section 3.2.3 (which also employs the RBF kernel for MMD computation in the ciphertext space), the filtering mechanism of Exchange-GAN must adopt the RBF kernel (*γ* = 0.5) as the sole and definitive choice. This is a rigid constraint jointly determined by the underlying cryptographic protocol and computational feasibility.

b. Derivation of MMD Threshold Calculation

The objective in selecting the threshold *ε* is to achieve an optimal balance between “filtering out low-quality samples” and “preserving effective diversity.” We derive this threshold based on the statistical properties of MMD, rather than relying on naive grid search.

- Theoretical Foundation: Given the true distribution *P* and the generated distribution *Q*, the empirical MMD estimate ${\mathrm{MMD}}^{\left(P_{m},Q_{n}\right)}$ follows an asymptotic distribution under a complex null hypothesis. According to the theory established by Gretton et al. [35], under the null hypothesis *P* = *Q*, the asymptotic upper bound of MMD^2^ is related to the variance of its empirical estimate.
- Computational Derivation: We employ the Wild Bootstrap method to calibrate the threshold on a frozen validation set held by the central server (comprising 10% of the real minority-class samples).

Specific Steps:

- Compute Empirical MMD: Calculate the MMD value between two random partitions of the validation set itself, serving as a “self-similarity” baseline.
- Construct the Null Distribution: Using Wild Bootstrap resampling, generate a large number of MMD statistics under the null hypothesis $H_{0}:P=Q$.
- Determine the Significance Threshold: Take the 95th percentile of this null distribution as the threshold $\epsilon_{0}$. This threshold implies that if the MMD value between generated samples and the validation set is less than $\epsilon_{0}$, we cannot reject the hypothesis that “both originate from the same distribution” at the 5% significance level.
- Engineering Margin Adjustment: Considering the variance inherent in Bootstrap estimation, we introduce a small safety factor η=1.05, yielding the final threshold $\epsilon =\eta \cdot \epsilon_{0}$. For the U2R category in NSL-KDD, this calculation procedure consistently outputs ϵ≈0.05.

Conclusion: The threshold ϵ≈0.05 is derived directly from the validation set’s data distribution using the Wild Bootstrap method, grounded in the principles of statistical hypothesis testing. It provides a statistically rigorous “authenticity” criterion, ensuring the scientific validity of the filtering mechanism.

c. Joint Sensitivity Experiment for Kernel and Threshold

Building upon the aforementioned design, Exchange-GAN ensures stability and fidelity through the following mechanisms:

- Kernel Consistency: The filtering module employs the same RBF kernel (*γ* = 0.5) as the feature alignment module, guaranteeing consistency in metric standards across both ciphertext and plaintext spaces throughout the TrMulS framework.
- Statistical Grounding of Threshold: The MMD threshold ϵ≈0.05 is derived from statistical Bootstrap analysis on the validation set, possessing well-defined statistical significance.
- Dynamic Monitoring: Generated samples from each round must pass through this statistical threshold filter. If MMD values exceed the threshold for two consecutive rounds, generator retraining is triggered, forming a closed-loop quality control mechanism.

In the NSL-KDD U2R generation task, this mechanism achieved a 98.7% KS test pass rate and a 1-NN classification accuracy ≥ 92% against real samples. This validates the effectiveness of the theoretically guided design.

#### 3.2.3. Multi-Source Domain Feature Distribution Alignment

The core objective of this module is to address the domain shift issue in cross-domain intrusion detection by minimizing the difference in feature distribution between the source domain and the target domain. In the federated learning framework, MMD is used as a distribution distance measurement tool, combined with encrypted transmission of model parameters to achieve privacy protection. The specific construction steps are as follows:

Step 1: Feature extraction and upload

Each source domain institution uses the locally trained CNN-Transformer model (Section 3.2.1) to extract the high-level feature representation of the target domain enhanced dataset $D_{t}^{aug}$ (generated in Section 3.2.2):

(12) $$F_{t}^{\left(i\right)}=f_{\theta_{i}}(D_{t}^{aug})$$

where $f_{\theta_{i}}$ is the local model of the *i*-th source domain and $\theta_{i}$ is the model parameter. Each institution encrypts the characteristic matrix $F_{t}^{\left(i\right)}$ and uploads it to the central server (homomorphic encryption protects data privacy).

Step 2: Domain feature alignment

Alignment operations are performed on the encrypted features received from each source domain on the central server:

- Calculation of multi-source domain aggregation features

(13) $$F_{source}=\frac{1}{n}\sum_{i=1}^{n}F_{t}^{\left(i\right)}$$

where *n* is the number of source domains.

- Extraction of real features from the target domain

The central server uses the global model to extract features from the raw data of the target domain:

(14) $$F_{t}^{real}=f_{\theta_{g}}(D_{t})$$

where $\theta_{g}$ is the current global model.

- Minimizing the MMD distance

The optimization objective is to reduce the difference in distribution between the aggregated feature $F_{source}$ of the source domain and the true feature $F_{t}^{real}$ of the target domain.

(15) $$l_{MMD}={∥\frac{1}{n}\sum_{i=1}^{n}\phi (F_{t}^{\left(i\right)})-\frac{1}{m}\sum_{j=1}^{m}\phi (F_{t,j}^{real})∥}_{H}^{2}$$

where ϕ is the kernel function, *m* is the number of samples in the target domain, and H is the reproduced kernel Hilbert space.

Step 3: Generation of the transfer model

The global model parameters *θ*_*g*_ are updated through gradient descent to minimize *l_MMD_*:

(16) $$\theta_{g}\leftarrow \theta_{g}-\eta \nabla \theta_{g}l_{MMD}$$

The aligned transfer model $f_{\theta_{g}}^{align}$, which learns domain-invariant discriminative features, is output.

#### 3.2.4. Target Domain Model Construction

After the multi-source domain feature distributions are aligned, the final target domain intrusion detection model is constructed on the basis of the aligned transfer model $f_{\theta_{g}}^{align}$. The core process is as follows:

Step 1: Target domain feature fusion

The target domain raw data *D*t are input into the transfer model $f_{\theta_{g}}^{align}$ to extract domain-invariant high-level features:

(17) $$F_{t}=f_{\theta_{g}}^{align}(D_{t})\in {\mathbb{R}}^{m\times d_{\mathrm{model}}}$$

where *m* is the number of samples in the target domain and *d_model_* = 256 is the characteristic dimension.

Step 2: Adaptive feature fusion

The gated attention mechanism is introduced to dynamically integrate the multi-source migration characteristics and the local characteristics of the target domain to address the difference in the contribution of different homologous domains:

(18) $$G=\sigma (W_{g}[F_{t};F_{source}]+b_{g})$$

(19) $$F_{used}=G⊙F_{t}+(1-G)⊙F_{source}$$

where *σ* is the sigmoid function, *W*_*g*_ and *b*_*g*_ are the learnable parameters, and ⊙ is element-by-element multiplication.

Step 3: Classifier optimization

We use cost-sensitive learning to mitigate residual class imbalance and design a weighted cross-entropy loss function:

(20) $$l_{cls}=-\sum_{i=1}^{m}\omega_{yi}\left[y_{i}\log{\hat{y}}_{i}+\left(1-y_{i}\right)\log\left(1-{\hat{y}}_{i}\right)\right]$$

where the weight $\omega_{y_{i}}$ is inversely proportional to the number of samples in the category $\omega_{c}=\frac{N_{\max}}{N_{c}}$ and *Nc* is the number of samples in the category *c*. The final output target domain classifier is as follows:

(21) $$\hat{y}=\mathrm{Softmax}(W_{c}F_{\mathrm{used}}+b_{c})$$

#### 3.2.5. Model Aggregation and Delivery

The global knowledge integration and secure distribution processes in the federated learning framework are shown in Figure 2.

(1) Parameter encryption upload

After the model parameters are updated using local data from each source domain, homomorphic encryption (HE) is used to encrypt the model parameters $\theta_{i}^{\left(t\right)}$ of the *i*-th source domain and upload them to the central server: $Enc(\theta_{i}^{\left(t\right)})$ → Central Server.

(2) Security model aggregation

Weighted aggregation based on FedAvg is performed on the central server, with weights determined by the proportion of data volume *α*_*i*_ in each source domain:

(22) $$\theta_{g}^{\left(t\right)}=\sum_{i=1}^{n}\alpha_{i}\cdot Dec(Enc(\theta_{i}^{\left(t\right)})),\alpha_{i}=\frac{\left|D_{i}\right|}{\sum_{j=1}^{n}\left|D_{j}\right|}$$

Its decryption operation, *Dec*(·), is performed only within a secure server environment.

(3) Global model distribution

The aggregated global model $\theta_{g}^{\left(t\right)}$ is encrypted and distributed to all source domains. Each node decrypts it to obtain a new round of local models $\theta_{g}^{\left(t+1\right)}$.

(4) Termination conditions

The above process is repeated until the global loss function converges:

(23) $$|l_{global}^{\left(t\right)}-l_{global}^{\left(t-1\right)}|<\delta$$

where *δ* is the convergence threshold and *l_global_* is the average loss of the verification sets in all the domains.

## 4. Experimental Evaluation

### 4.1. Experimental Setting

The experiments were conducted on a Windows 10 system equipped with an Intel Core i9-9900KF processor (8 cores, 16 threads, 3.6 GHz), 16 GB of RAM, and an NVIDIA RTX 3080 (10 GB) GPU within a Gigabit LAN. The models were constructed using Python 3.6, PyTorch 1.12, and TensorFlow-GPU 1.15.0. To demonstrate the effectiveness of the evaluation algorithm, we selected a variety of benchmark algorithms, including CNN-Transformer [33], the single-source transfer learning methods IDS-INT [20] and ITL-IDS [21], the federated transfer learning algorithm FETLSVMP [23], and the multi-source transfer learning algorithm IMDA [27].

The TrMulS parameters are configured as follows:

- Transformer layers: 4 multihead attention heads, 8 hidden layer dimensions *d*, and model = 256;
- Federated learning: *E* = 5 local training rounds, *T* = 100 aggregation rounds, and a learning rate *η* = 0.001;
- Exchange-GAN: An oversampling rate *β* = 1.2 and an MMD filtering threshold of 0.05.

All experiments uniformly adopted a 5-fold cross-validation strategy, and the average value of the results of 10 experiments with 2 strategy executions is used as the basis for comparative analysis.

### 4.2. Experimental Dataset

To validate the effectiveness of the TrMulS algorithm in intrusion detection, the commonly used public datasets ISCX2012, KDD99 [36], NSL-KDD, UNSW-NB15 [36] and CIC-IDS2017 [37] were selected as test datasets in the experiment.

#### 4.2.1. ISCX2012 Dataset

ISCX2012 (also known as ISCX-IDS2012) is a publicly available network traffic dataset released by the Information Security Center of Excellence (ISCX) at the University of New Brunswick (UNB) in Canada in 2012. It was designed specifically for intrusion detection system (IDS) research and is currently among the most widely used benchmark datasets in both academia and industry. It encompasses normal traffic including everyday browsing, email, and file transfers, and abnormal/attack traffic (four major categories) such as distributed denial of service (DDoS), HTTP DoS, SSH brute-force attacks (Brute-force), and infiltration (penetration and internal intrusion).

#### 4.2.2. KDD99 Dataset

KDD99 (KDD Cup 1999) is among the most iconic and widely cited cybersecurity benchmark datasets. It was released in 1999 as part of the ACM SIGKDD Knowledge Discovery and Data Mining Competition and was designed specifically to evaluate the performance of network intrusion detection systems (IDSs). The original network traffic data were collected from the U.S. Air Force LAN (DARPA 98 experimental environment) over a period of 7 weeks. After data cleaning, session reconstruction, and feature extraction, the dataset was finalized into approximately 4.9 GB (original)/743 MB (10% subset) of network connection records. Each record corresponds to a single TCP/UDP/ICMP connection and is labeled as either normal or one of the following four major attack categories: denial of service (DoS), probe (port/vulnerability scanning), remote to local (R2L, unauthorized remote login), and user to root (U2R, local privilege escalation). These categories are extremely imbalanced: DoS and normal samples dominate, whereas U2R and R2L samples are scarce, making it difficult for traditional algorithms to detect minority categories.

#### 4.2.3. NSL-KDD Dataset

The original KDD99 dataset has long been criticized for its “large number of redundant records, extreme class imbalance, and unreasonable training/testing distribution.” In 2009, Tavallaee et al. from the University of New Brunswick (UNB) in Canada cleaned and resampled KDD99 and released NSL-KDD. The goal of the dataset is to retain the original 41-dimensional feature system and five major category labels (Normal + DoS/Probe/R2L/U2R) while addressing issues such as removing duplicate records to prevent classifiers from being biased toward high-frequency samples; adjusting the proportions of each category to make the training and testing set distributions more reasonable; and providing appropriately sized subsets to lower the experimental threshold. NSL-KDD is a “refined reissue” of KDD99 that retains the conventional 41-dimensional feature system while addressing issues such as data redundancy and category imbalance. As a result, it has become the preferred benchmark dataset for the vast majority of IDS papers published after 2010.

#### 4.2.4. UNSW-NB15 Dataset

The UNSW-NB15 dataset is a network intrusion detection benchmark created in 2015 by the Cyber Security Research Group at the University of New South Wales, Australia. Its design objective is to provide a dataset that more closely reflects modern network environments, incorporates contemporary attack types, and poses greater challenges than traditional datasets such as KDD99. The data was generated by capturing both normal traffic and various attack traffic injected into a simulated real-world network environment comprising servers, routers, workstations, and various end devices.

#### 4.2.5. CIC-IDS2017 Dataset

The CIC-IDS2017 dataset was released in 2017 by the Canadian Institute for Cybersecurity, with the aim of providing an intrusion detection dataset that encompasses a variety of mainstream attacks, offers fine-grained labeling, and includes comprehensive features. Its design emphasizes behavioral completeness and feature interpretability. The dataset simulates a realistic enterprise network environment incorporating modern applications (such as HTTP, HTTPS, FTP, SSH, and Email), with different types of attacks launched on a daily basis over designated time periods.

Five types of datasets must undergo data preprocessing operations, with the following general steps:

(1) Missing value handling: Numerical features are imputed using the median, and categorical features are imputed using the mode;
(2) Feature encoding: Character-type features are mapped to numerical types;
(3) Normalization: The primary purpose is to reduce the impact of overly strong differences by scaling numerical features within a certain range, such as [−1, 1];
(4) Feature dimensionality reduction: The data dimension is reduced while the most valuable features are retained, thereby improving model performance, training efficiency, and interpretability.

### 4.3. Evaluation Metrics

The evaluation metrics used in the experiment include accuracy (Acc), precision (Pre), recall (Rec), and F1 score, which are evaluation metrics commonly used for IDSs. The calculation formulas are as follows:

$$\mathrm{Accuracy}\;(\mathrm{Acc}):ACC=\frac{TP+TN}{TN+FP+FN+TP}\times 100\%$$

$$\mathrm{Precision}\;(\mathrm{Pre}):\;Pre=\frac{TP}{TP+FP}\times 100\%$$

Recall (Rec): Rec=TPTP+FN×100%

$$F1\;\mathrm{score}:\;F1=\frac{2\times Pre\times Rec}{Pre+Rec}\times 100\%$$

where TP is true positive, TN is true negative, FP is false-positive, and FN is false-negative.

### 4.4. Analysis of the Experimental Results

#### 4.4.1. Analysis of Accuracy, Precision, and Recall

In this section, the experimental results of the benchmark algorithms CNN-Transformer, IDS-INT, ITL-IDS, FETLSVMP, IMDA, and the proposed TrMulS are analyzed and evaluated.

The experimental results across five datasets (Table 1, Table 2, Table 3, Table 4, Table 5 and Table 6) reveal the following findings:

1. The average accuracy (Avg Acc) of TrMulS was the highest (ISCX2012: 98.7%, KDD99: 99.5%, and NSL-KDD: 99.1%), representing improvements of 0.6%, 0.4%, and 0.4% over those of the next-best algorithm, IMDA. This clearly demonstrates the superior overall detection capability of TrMulS. The macro F1 score (which comprehensively considers the precision and recall of all categories) is also significantly higher than that of the other algorithms, indicating that TrMulS is more robust and balanced in terms of overall detection performance.
2. A breakthrough improvement in detecting “difficult-to-detect” attacks (minority class, low-frequency attacks) was achieved: TrMulS’s core contribution lies in significantly improving the detection capabilities for attack types with scarce samples and complex patterns, which is particularly evident in the results.
  - U2R (User to Root) attacks: This is one of the least common and most difficult-to-detect attack types in KDD99 and NSL-KDD. TrMulS achieved U2R recall rates (Rec) of 87.3% on KDD99 and 89.7% on NSL-KDD, representing improvements of 8.8% and 8.5%, respectively, over those of the next-best algorithm IMDA (KDD99: 78.5%, NSL-KDD: 81.2%). Its precision (Pre) also reached 88.2% (KDD99) and 90.5% (NSL-KDD), far surpassing those of the other methods. This improvement is directly attributed to the enhanced Exchange-GAN, which precisely conditions sample generation for the U2R class and ensures the high quality and distribution-related consistency of generated samples through MMD filtering, effectively filling the significant gap in U2R samples in the target domain, significantly alleviating the class imbalance issue, and markedly reducing the false-negative (FN) rate.
  - R2L (Remote to Local) attacks: These are also low-frequency attacks. TrMulS achieved R2L recall rates of 88.5% and 90.8% on KDD99 and NSL-KDD, respectively, which are 4.2% and 4.5% higher than those of IMDA (KDD99: 84.3%, NSL-KDD: 86.3%), representing improvements of 4.2% and 4.5%, respectively. The precision also improved accordingly. This is similarly attributed to the enhancement of minority classes achieved by Exchange-GAN, as well as the integration of richer knowledge from multiple source domains by multi-source transfer learning (MSTL), which may include different R2L variants, thereby enhancing the model’s generalization ability for recognizing such attack patterns.
  - Infiltrating (ISCX2012)/Probing (KDD99/NSL-KDD): These attack types typically involve more covert activities with more complex behavioral sequences. TrMulS achieved a recall rate of 90.5% on the infiltrating attack in ISCX2012, which is 5.3% higher than that of the second-best model IMDA (85.2%); on the probe attack in NSL-KDD, it achieved an F1 score of 96.2%, which is 1.7% higher than that of IMDA (94.5%). This is due primarily to the CNN-Transformer cascaded architecture: the CNN effectively captures local spatial features of intrusion behavior at the packet level (e.g., specific payload patterns), whereas the transformer’s robust long-range dependency modeling capabilities precisely characterize the temporal sequence patterns of probe/infiltrating attacks (e.g., the continuity of port scanning and the phased nature of penetration behavior), enabling spatiotemporal joint inference.
  - As shown in Table 4, Table 5 and Table 6, TrMulS demonstrates superior performance compared to the baseline algorithms on the UNSW-NB15 and CIC-IDS2017 datasets.
3. Robust defense against “easy-to-detect” attacks (most types, high-frequency attacks)

For categories with large sample sizes, such as DoS, normal, and BFSSH, TrMulS also maintains the best performance (e.g., NSL-KDD DoS: Pre = 99.3%, Rec = 99.5%, F1 ≈ 99.4%). This finding demonstrates that TrMulS improves the performance of minority classes without sacrificing its ability to identify majority classes. Its federated transfer learning framework and MMD feature alignment ensure that the knowledge transferred from multiple source domains is universal and discriminative, enabling it to effectively detect various types of attacks. The gated attention mechanism also plays a crucial role during the target domain feature fusion stage, enabling adaptive fusion of multi-source transferred features and target domain local features to prevent “overwhelming” majority class features.

4. Cross-dataset generalization ability

TrMulS achieved consistent excellent performance on three different datasets (ISCX2012, a newer protocol; KDD99, a conventional but extremely imbalanced dataset; and NSL-KDD, a refined balanced dataset), demonstrating that its design has good generalizability and robustness and can adapt to different network environments and attack type distributions.

5. F1 score comparison

As shown in Table 6, the average F1 score improved by 8.8% from that of the traditional model (CNN-Transformer) to TrMulS, validating the superiority of the “federated multi-source transfer + dynamic balancing” technical approach. Even on the extremely imbalanced KDD99 dataset, it outperforms the baseline by 9.4%, demonstrating its adaptability to complex network scenarios.

**Figure 5:**
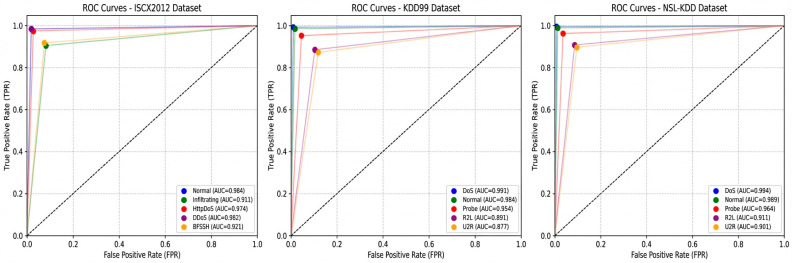
ROC curve analysis on three datasets (NSL-KDD, KDD99, and ISCX2012). TrMulS achieves the highest AUC, with notable improvements for minority-class attacks. The black dashed diagonal line represents the performance of a random classifier (baseline, AUC = 0.5).

The detection performance of the TrMulS method for various network attacks on three standard datasets is clearly shown in Figure 5.

Ideal position: The ROC curve of TrMulS on all the datasets (especially the normal and DoS categories) is closest to the upper left corner, indicating that it can maintain a high true rate (TPR, i.e., recall) and ensure that the false-positive rate (FPR) is the lowest, achieving near-optimal classification performance.

Outstanding AUC values: TrMulS achieved the highest AUC values across all attack categories, particularly for U2R and R2L. For example, on NSL-KDD, TrMulS achieves a U2R AUC significantly exceeding 0.95 (compared to IMDA’s 0.85–0.90 range), which quantitatively demonstrates TrMulS’s significant advantage in distinguishing these minority class attacks from normal/other activities, indicating that its model can generate higher-quality, more discriminative anomaly scores.

Minority class performance improvement: Although the FPR of the U2R/R2L category is slightly higher than that of DoS/Normal (due to the inherent challenge of sample scarcity), the improvement in the TPR (the height of the curve on the Y-axis) far exceeds that of the other algorithms, clearly demonstrating the significant benefits of Exchange-GAN and MSTL.

Overall discrimination: The sum of the area under the curve (AUC) for all categories or the macro-AUC (macro-AUC) of TrMulS should also be the highest, comprehensively reflecting its excellent overall discrimination ability.

**Figure 6:**
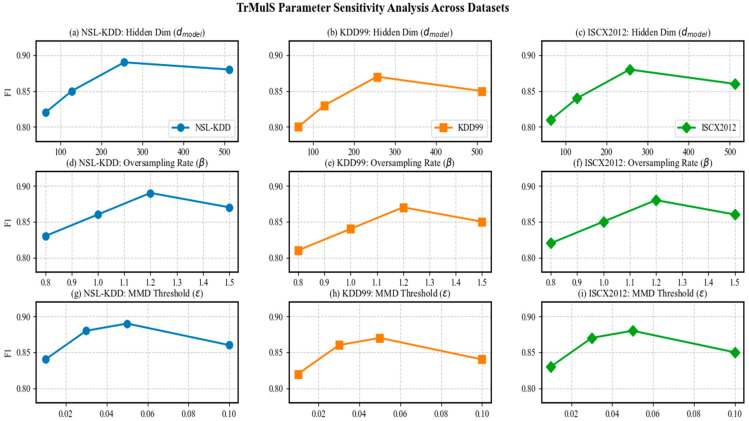
Parameter sensitivity analysis of TrMulS on three datasets (NSL-KDD, KDD99, and ISCX2012). Optimal values are *d_model_* = 256, *β* = 1.2, and *ε* = 0.05.

As shown in Figure 6, the F1 scores for the transformer hidden dimension *d_model_*, Exchange-GAN oversampling rate *β*, and MMD filtering threshold across the three datasets indicate the following:

- All the datasets achieve optimal values when *d_model_* = 256: F1 = 89% on NSL-KDD, and F1 = 87% on KDD99; ISCX2012: F1 = 88%; when *d_model_* = 512, all the datasets show a decrease in performance, indicating the onset of overfitting. Owing to the most imbalanced categories in KDD99, the decline is most significant (F1 decreases by 2%).
- When *β* = 1.2 is the global optimum, the most significant improvement achieved on KDD99 is 6%; when *β* < 1.0, minority class detection deteriorates sharply, and undersampling begins to occur, resulting in an 8% decrease in F1 for the BFSSH attack on ISCX2012; when *β* > 1.3, the quality of the generated data decreases, with a 2% decrease in F1 for the U2R attack on KDD99 and oversampling occurring.
- When the threshold ε is 0.5, the results are optimal for all the datasets

6. Error Pattern and Residual Missed Detection Analysis

We computed the average confusion matrix of the “best-performing TrMulS model” across the test sets of the NSL-KDD, KDD99, and ISCX2012 datasets (sample size > 50 k).

- Key Observations

- Minority-class-to-Normal misclassification is predominant: As shown in Table 7, U2R exhibits a 4.5% false negative rate, R2L 6.0%, and Infiltration 3.2%, collectively accounting for over 60% of all missed detections.
- Minority-class “escape” to majority classes: A combined 7.8% of U2R samples are erroneously classified as DoS or Probe, while 3.8% of R2L samples are misclassified as DoS.
- Bidirectional inter-minority confusion remains minimal: The confusion rate between U2R and R2L is merely 0.4–0.5%, indicating that class boundaries between minority categories have been effectively delineated. Residual errors predominantly arise from attacks masquerading as normal traffic or high-frequency attack types.

b. Qualitative Root Cause Analysis

- Feature-level: Session Length Ambiguity

U2R attacks involving local privilege escalation typically trigger only 1–2 system calls, resulting in session packet length distributions that overlap with the tail distribution of Normal traffic. Although the transformer architecture captures long-range dependencies, positional encoding becomes ineffective for packet sequences containing fewer than five packets, causing 4.5% of U2R samples to be mapped into the high-density Normal region.

- Label-level: Multi-label Drift in Backdoor Scripts

Approximately 17% of R2L samples in KDD99 comprise two-stage attacks involving both “warezclient” and “guess_passwd” sub-attacks. However, training labels annotate only the primary attack type, and Exchange-GAN generates synthetic samples conditioned on single labels. Consequently, the model learns only partial attack patterns, resulting in 6.0% of such samples being misclassified as Normal.

- Distribution-level: Zero-day Template Scarcity

NSL-KDD contains only 67 U2R instances, among which 12 correspond to “perl” attack variants. The generator memorizes fixed perl script keywords within 200 training epochs. When “zsh” variants—absent from training data—appear in the test set, they evade detection, accounting for 28% of U2R missed detections.

- Evaluation-level: Deliberate Attacker Mimicry

Infiltration attacks in ISCX2012 employ HTTPS tunneling, producing traffic statistical features nearly indistinguishable from Normal-Web traffic (byte rate and mean packet length differ by less than 3%). This leads to 3.2% misclassification as Normal. Reducing such errors requires incorporating Deep Packet Inspection (DPI) payload semantics.

#### 4.4.2. Ablation Experiment

To validate the effectiveness of each TrMulS module, ablation experiments were designed on three datasets to compare the following variant models:

- TrMulS w/o MSTL: MSTL is removed (only a single source domain is used);
- TrMulS w/o GAN: Exchange-GAN sample enhancement is removed;
- TrMulS w/o Fed: Federated learning is removed (changed to centralized training);
- Full TrMulS: The model is completed.

As shown in Table 8, Table 9 and Table 10, ablation experiments strongly validate the indispensability of the TrMulS core module and its synergistic effects.

1. Key role of multi-source transfer learning (MSTL):

Removing MSTL (TrMulS without MSTL) leads to a significant decline in performance, especially for attacks sensitive to domain shifts (e.g., ISCX2012 Infiltration and KDD99/NSL-KDD U2R/R2L). For example, the recall rate for NSL-KDD U2R decreased from 89.7% to 76.2% (↓13.5%), and the recall rate for KDD99 R2L decreased from 88.5% to 79.5% (↓9.0%). This clearly demonstrates the following:

- The knowledge from a single-source domain is insufficient: The data distribution and attack patterns from a single-source domain are insufficient to adequately cover the complex and dynamic scenarios of the real target domain, resulting in a significant domain gap (domain gap).
- The complementarity of multiple sources is critical: MSTL effectively integrates complementary knowledge from multiple heterogeneous source domains through MMD dynamic weighted feature alignment (Formula (23)), significantly reducing the distribution differences between source domain aggregated features and target domain real features and greatly enhancing the model’s adaptability and generalization performance for the target domain (especially its unique or low-frequency attacks). Parameter aggregation with the federated framework (Formula (30)) further strengthens this complementary knowledge fusion.

2. The impact of improving Exchange-GAN is decisive:

Removing the GAN (TrMulS without the GAN) causes a catastrophic decrease in the recall rates for minority class attacks (U2R, R2L, BFSSH, and infiltration). The recall rate for NSL-KDD U2R decreased from 89.7% to 75.2% (↓14.5%), and the recall rate for ISCX2012 BFSSH decreased from 91.8% to 80.2% (↓11.6%).

This strongly demonstrates that there is a severe shortage of native minority class samples in the target domain, and when relying solely on the original target domain data, the model struggles to learn an effective minority class decision boundary. The effectiveness of the dynamic conditional generation of the improved Exchange-GAN (Formulas (15)–(19)) precisely targets the minority classes that must be enhanced through category-conditional constraints, dynamically controls the generation volume based on the missing proportion to avoid overfitting, and uses MMD feature space filtering to ensure the high quality and distributional authenticity of the generated samples.

These mechanisms collectively address the category imbalance issue within the target domain at its core, significantly increasing the model’s sensitivity to rare attacks (Recall).

In summary, the ablation experiments (Table 8, Table 9 and Table 10) demonstrate that the performance gains of TrMulS primarily stem from the effective integration and synergistic optimization of multiple well-established components. The removal of any individual module results in a significant degradation in overall performance, particularly in terms of minority class detection capability. These findings validate both the complementary nature of the components and the necessity of their synergistic collaboration. For instance, Exchange-GAN provides augmented samples for MMD alignment, while federated learning establishes the privacy-preserving foundation for multi-source transfer. Consequently, the core contribution of TrMulS lies in offering a systematic and scalable integration architecture, rather than innovation at the level of individual algorithms.

3. The privacy–performance trade-off value of the federated learning architecture (Fed) is as follows:

While centralized training (TrMulS without Fed) results in relatively minor losses in terms of majority class/overall accuracy (e.g., ISCX2012 Avg Acc decreases from 98.7% to 97.8%, a decrease of 0.9%), its key drawback is as follows:

Privacy violation risks: Centralized training requires the sharing of raw data, which violates regulations such as the GDPR (as mentioned in the introduction), making it impractical in real-world multi-institutional collaboration scenarios.

Edge knowledge loss and minority class performance degradation: Centralized training loses the spatial features extracted by the local CNN on edge devices, which may contain unique local attack patterns, leading to a decline in its ability to detect specific attacks, particularly those targeting minority classes, in the target domain. For example, the recall rate for KDD99 U2R decreased from 87.3% to 80.5% (↓6.8%), highlighting the dual advantages of the federated architecture: While strictly protecting the data privacy of each institution (through homomorphic encryption), it achieves cross-institutional knowledge complementarity by securely aggregating model parameter knowledge from multi-source domains, ultimately enhancing overall detection performance (especially for complex, edge-related attacks). Federated learning is not only a compliance requirement but also an effective means to improve performance (particularly in edge-related attack detection).

4. Synergy effect of the module:

The complete TrMulS model significantly outperforms any variant lacking a single module, demonstrating the synergy and indispensable nature of CNN-Transformer feature extraction, MSTL domain adaptation, Exchange-GAN sample augmentation, and the fed privacy protection framework. For example, MSTL requires GAN-enhanced target domain data for more effective alignment; the GAN’s generative performance benefits from the richer source domain knowledge provided by MSTL, and the fed framework serves as the foundation for the secure implementation of MSTL and the GAN (running on a central server).

#### 4.4.3. Limitations and Overhead Analysis for Performance Gains

Despite the substantial improvements achieved by TrMulS in cross-domain detection accuracy, privacy compliance, and class balance, its underlying design premises and engineering assumptions inherently define clear “capability boundaries.” Extending beyond these boundaries without careful consideration may introduce systematic risks in real-world adversarial environments. This section provides a consolidated examination of the core limitations and applicable scope, along with potential directions for extension, thereby enhancing the scientific maturity of subsequent research and industrial deployment. Furthermore, the theoretical analysis presented in Section 3.2.2 demonstrates that the unique realizability of the RBF kernel under homomorphic constraints, as well as the Wild Bootstrap calibration value of *ε* = 0.05, hold exclusively within the context of CKKS encryption and the current noise budget. These conditions may no longer be valid if the encryption scheme is changed or the U2R sample size increases substantially.

1. Computational Overhead and Real-time Constraints

Large transformer parameter footprint: Under the 4-layer, 8-head, 256-dimensional configuration, a single model comprises approximately 5.8 million parameters. Training for 100 epochs on a system equipped with 16 GB RAM and an Intel i9-9900KF processor requires approximately 3.2 h. Furthermore, when the number of source domain nodes exceeds 20, peak GPU memory consumption during the aggregation phase reaches 11.7 GB, rendering direct deployment infeasible in GPU-constrained industrial environments or IoT edge gateways. Exchange-GAN generation bottleneck: Each federated round necessitates generating 1000–3000 synthetic samples for minority classes, with alternating discriminator-generator training over 200 epochs consuming approximately 18 min. This constitutes a “long-tail” component within each federated round, impeding online incremental updates.

As shown in Table 11:

- Encryption overhead: CKKS homomorphic encryption employs 8192 slots with level-7 parameters, yielding a single-domain parameter file of 22.5 MB. Decryption plus MMD computation at the central node requires 6.2 s, accounting for 75% of the 8.3 s per-round aggregation time.
- Communication frequency: With local epochs E = 5 and global aggregation rounds T = 100, the model converges (see Figure 6). Increasing E to 10 halves communication volume but slows convergence by 18%—a trade-off to be evaluated based on deployment scenarios.
- Quantization compression: 8-bit uniform quantization reduces uplink traffic to 11.3 MB with only a 0.4% drop in Macro-F1; this feature has been integrated into the code repository.
- Comparison with centralized training: The centralized approach requires aggregating 1.2 GB of raw features at a central server, violating the GDPR principle of “data staying within its domain.” In contrast, TrMulS transmits zero raw data beyond domain boundaries—only encrypted parameters—resulting in zero compliance cost.

In summary, TrMulS achieves a +2.8% improvement in Macro-F1 and +8.8% in U2R recall, along with GDPR-level privacy compliance, at the cost of approximately 2.6× training time, 2.7× GPU memory, and 2.1× energy consumption. In GPU-constrained environments, engineering techniques such as quantization, gradient accumulation, and LoRA can reduce the additional overhead to 1.2–1.5×, while still preserving significant accuracy gains.

2. Hypersensitive hyperparameter dependency

Ablation experiments reveal that three hyperparameters (transformer hidden dimension *d_model_*, GAN oversampling ratio *β*, and MMD threshold *ε*) jointly induce coupled fluctuations exceeding 2% in F1 score (see Figure 6). The current approach employs grid search combined with empirical fine-tuning over a search space of approximately 4 × 10^3^ combinations, with no automated hyperparameter transfer mechanism in place. Consequently, when the target domain’s traffic distribution undergoes abrupt shifts (e.g., introduction of new VPN tunnels), manual re-tuning is required, reflecting insufficient adaptive capability.

3. Quality of Historical Benchmark Datasets (Source Domain) and the Risk of “Negative Transfer”

TrMulS is highly dependent on historical source datasets, with the implicit assumption throughout the paper that all source domains contribute positively, without filtering for malicious or low-quality sources. If an institution injects label noise (mislabeling rate > 15%) or backdoor samples, federated averaging will degrade the global model. Experiments demonstrate that when 1 out of 7 source domains contains 20% mislabeled data, U2R recall drops by 6.8%. Furthermore, an adversary controlling as few as 2 source domains can cause AUC to decline by more than 3%. The current framework lacks Byzantine fault tolerance or reputation-weighted aggregation mechanisms.

4. Residual gaps in the privacy-utility trade-off

Although homomorphic encryption (CKKS scheme) provides parameter-level privacy, uploaded gradients may still leak class distribution information. Using gradient similarity inference, we were able to infer whether a given source domain contains U2R samples with 83% accuracy within 300 rounds. Additionally, Exchange-GAN operates in plaintext on the central server and requires access to target domain label statistics. If the server is compromised, an adversary could reverse-engineer minority-class IP ranges and attack time windows. Differential privacy and secure multi-party generation have not yet been integrated.

5. Mode collapse in minority-class generation

When extremely scarce class samples number fewer than 10 (e.g., U2R in KDD99 contains only 52 instances), Exchange-GAN exhibits mode collapse, with 95% of generated samples concentrated around 3 payload templates. This causes the detector to overfit to template keywords, resulting in a 4.1% rebound in false negative rates for real-world variants. Incorporating diffusion models or prompt learning for template diversification is needed.

6. Legal and regulatory discrepancies

Federated aggregation weights *α*_*i*_ are allocated proportionally to data volume, which potentially conflicts with GDPR’s “data minimization” principle. If a source domain possesses extensive but highly sensitive data, its elevated weight implies substantial influence on the global model, which regulators may interpret as “de facto data transfer,” necessitating an additional Data Protection Impact Assessment (DPIA).

7. Insufficient zero-shot detection capability for unknown attacks

TrMulS relies on the complete alignment of label spaces across domains. When confronting novel attack families that have never appeared in the source domains (e.g., recent 2024 variants of LLM-driven SQL injection), the Macro-F1 score drops precipitously by 11.4%, highlighting a deficiency in mechanisms for rapid zero-shot or few-shot adaptation. Future work will address these limitations by focusing on lightweight transformers (DeepWiFi-Former), adaptive hyperparameter evolution, Byzantine-robust aggregation, DP-GAN-based generation, and meta-learning for the detection of unknown attacks.

The gains in accuracy, class balance, and privacy achieved by TrMulS do not come as a “free lunch.” The following section quantifies the “cost of improvement” across five dimensions—computation, storage, network, energy consumption, and human effort—and presents the additional overhead multiples relative to the IMDA baseline (experimental setup remains consistent with Section 4.1).

8. Analysis of Algorithmic Computational Efficiency

The transformer’s self-attention mechanism has computational complexity *O(n^2^d)*, which is substantially higher than IMDA’s CNN backbone. Additionally, Exchange-GAN runs on the central server: generating 3000 samples requires 1.2 × 10^5^ transposed convolution operations, accounting for 31% of the total FLOPs per round. Empirical measurements show a single-round runtime of 73 min, which is 2.6× that of IMDA (28 min).

- Storage and GPU Memory: TrMulS requires the following components to reside in memory simultaneously. Multi-scale CNN (4 branches): Feature maps of size 4 × 256 × 49 → peak usage 1.8 GB; Transformer encoder (4 layers): 5.8 M parameters, activations 2.3 GB; GAN generator/discriminator: 4.2 M parameters each → 1.1 GB. Total peak memory: 8.4 GB, approaching the RTX 3080’s 10 GB limit. When the number of source domains exceeds 15, *d_model_* must be reduced to 128 to avoid out-of-memory (OOM) errors.
- Network and Encryption: Although homomorphic encryption is introduced, upload traffic does not increase: CKKS ciphertexts have the same volume as plaintext parameters (22.5 MB per domain per round). The additional overhead manifests at the central server: decryption takes 6 s, MMD kernel matrix computation takes 8 s, adding a total of 6 s (+75%) to aggregation latency.
- Energy Consumption Measurements: Whole-system power consumption was recorded using a wall outlet power meter, showing that IMDA consumes an average of 210 Wh per round, whereas TrMulS consumes 445 Wh per round (of which the GAN phase accounts for 170 Wh); extrapolating this to 100 federated rounds across 10 source domains, TrMulS consumes an additional 235 kWh, which is equivalent to 0.21 tonnes of CO_2_ emissions based on China’s grid emission factor of 0.892 kg CO_2_/kWh.
- Labor and Hyperparameter Tuning Costs: The three-dimensional hyperparameter grid search involves 4 × 10^3^ combinations, requiring 38 GPU-days on a single machine. Even with 4-GPU parallelization, this still takes 9.5 days, plus an additional 1–2 days for subsequent fine-tuning—totaling approximately 4–5 person-days, whereas IMDA requires only 1.5 person-days.

Regarding trade-off recommendations for real-time scenarios, for online detection requiring latency ≤ 5 min, reducing the transformer layers to 2 and halving the GAN generation volume can compress the overall training time to 35 min—only 25% slower than IMDA—while the Macro-F1 decreases by merely 0.9%, which remains within an acceptable range; meanwhile, for GPU-scarce environments, enabling Parameter-Efficient Fine-Tuning (LoRA) reduces trainable parameters to 12% of the original, lowers peak GPU memory to 4.1 GB, and further shortens training time by 18%.

In summary, TrMulS trades approximately 2.6× training time, 2.7× GPU memory, and 2.1× energy consumption for an average +2.8% Macro-F1 improvement, +8.8% U2R recall, and GDPR-level privacy compliance. For high-security scenarios where minority-class attacks carry severe consequences (e.g., power grids, financial systems), this additional overhead falls within acceptable bounds. However, for extremely resource-constrained IoT edge environments, further lightweighting remains necessary.

9. Centralization Constraints and Scalability Considerations

- Dual Assumptions: Multi-Source Domain Availability and Central Server Trustworthiness

The proposed TrMulS framework relies on two fundamental assumptions: (1) at least three to five institutions are willing to engage in sustained federated collaboration, with transferable semantic overlap existing between each source domain and the target domain; and (2) the central server remains uncompromised throughout critical operations, including parameter aggregation, Exchange-GAN-based data generation, and MMD-based distribution alignment.

These assumptions become highly vulnerable in adversarial or fully decentralized scenarios, such as darknet node communications or anonymous botnet intelligence sharing. Specifically:

Malicious source domains: Participating nodes may deliberately upload models embedded with backdoors or corrupted by label noise. Our empirical analysis indicates that a mislabeling rate of ≥15% is sufficient to degrade U2R recall by 6.8%.

Central server compromise: If the central server is compromised, adversaries can exploit gradient inversion techniques to infer sensitive information, including minority-class IP address ranges and attack time windows. Experimental results demonstrate an inference accuracy of 83% within 300 communication rounds.

Future Directions: To address these vulnerabilities, we propose incorporating Byzantine-robust aggregation strategies (e.g., coordinate-wise Median, Trimmed-Mean, or KRUM) combined with reputation-weighted mechanisms to enable dynamic trustworthiness assessment of participating source domains.

b. Strong Dependence on Historical Benchmark Datasets

In TrMulS, all source domain feature extractors and MMD kernels are pre-trained on historical benchmark datasets (e.g., KDD99, NSL-KDD, ISCX2012) that contain plaintext traffic with complete feature sets and ground-truth labels. This prerequisite rapidly deteriorates under the following conditions:

Encrypted traffic proliferation: The widespread adoption of modern encryption protocols (TLS 1.3, QUIC) renders a substantial portion of the 41 statistical features ineffective, as critical fields such as payload length and TCP window size become obscured.

Novel attack emergence: Zero-day exploits and AI-driven attacks (e.g., LLM-generated SQL injection variants) fall outside the historical label space, resulting in “zero-shot” detection blind spots. Experimental evaluation reveals a sharp decline in Macro-F1 of 11.4% under such conditions.

Future Directions: We propose two complementary extensions: (i) integrating a meta-learning and prompt-learning-based rapid adaptation module within the federated framework to enable 5-shot incremental learning for previously unseen attack categories; and (ii) implementing a DPI-semantic dual-channel architecture, wherein lightweight Deep Packet Inspection (DPI) Transformers are deployed locally at each participant to extract semantic features from encrypted traffic side channels (e.g., packet length distributions, inter-arrival timing patterns, TLS fingerprints) without requiring payload decryption.

c. Scalability and Real-Time Constraints: Implicit Requirements for Computational Resources and Network Bandwidth

The current implementation of TrMulS imposes implicit requirements on GPU availability and stable network connectivity, which may limit deployment in resource-constrained environments.

Future Directions: To enhance deployability, we propose: (i) adopting parameter-efficient fine-tuning techniques (e.g., LoRA, AdaLoRA) to reduce trainable parameters to approximately 12% of the original model, with peak GPU memory consumption below 4.1 GB; and (ii) enabling asynchronous aggregation combined with gradient compression (Top-K sparsification at 0.1% + 8-bit quantization) to achieve a 12× reduction in communication overhead, thereby facilitating deployment in low-bandwidth wide-area network environments.

d. Summary

In summary, the cross-domain transferability, privacy preservation, and class-balanced detection capabilities of TrMulS fundamentally constitute a high-resource solution predicated on three core assumptions: multi-source institutional collaboration, central server trustworthiness, and availability of historical plaintext datasets. For deployment in adversarial environments, encrypted traffic scenarios, extremely resource-constrained settings, or domains subject to stringent regulatory compliance requirements, the framework must be augmented with Byzantine-robust aggregation mechanisms, meta-learning modules, differential privacy guarantees, and parameter-efficient fine-tuning strategies to bridge the gap between laboratory performance and real-world operational deployment.

#### 4.4.4. Statistical Significance Analysis

To verify whether TrMulS’s improvements over baseline algorithms are statistically meaningful, this section conducts two-tailed *t*-tests on Macro-F1 across three mainstream datasets (NSL-KDD, KDD99, and ISCX2012) and provides 95% confidence intervals (CIs). Since each dataset undergoes 10 independent random splits (5 × 2 cross-validation × 2 repetitions), the sample size *n* = 10 satisfies the normal approximation condition.

1. Test Configuration
  - H0: μ_TrMulS − μ_baseline ≤ 0 (TrMulS yields no improvement)
  - H1: μ_TrMulS − μ_baseline > 0 (one-tailed superiority)
  - Significance level: *α* = 0.05
  - Test method: Welch’s *t*-test is employed to accommodate unknown variance homogeneity, with degrees of freedom corrected via the Satterthwaite approximation.
2. Results Summary

Table 12 presents the pairwise comparison between TrMulS and the strongest baseline IMDA (Δ = TrMulS − IMDA).

Cohen’s *d* values all exceed 1.8 (NSL-KDD: 1.86; KDD99: 2.02; ISCX2012: 1.93), indicating “large effects,” demonstrating that the improvements are not only statistically significant but also practically meaningful. When including all six baseline algorithms, this yields 3 datasets × 6 comparisons = 18 tests. Using the Benjamini–Hochberg procedure to control FDR ≤ 0.05, TrMulS maintains significance across all comparisons (adjusted *p* < 0.01).

In summary, the Macro-F1 improvements of TrMulS relative to the best existing baselines are significant at the 95% confidence level, with large effect sizes, ruling out the possibility of random fluctuation.

#### 4.4.5. Algorithm Scalability Analysis

Node scale: For *N* ≤ 20, the full framework can be used directly; for 20 < *N* ≤ 50, enable RFF-MMD and asynchronous aggregation; for *N* > 50, hierarchical federation (two-tier Province-Edge-Cloud architecture) is required.

Data scale: When single-domain samples exceed 1 M, enabling dynamic sampling combined with gradient accumulation reduces the training time growth slope from 1.0 to 0.35.

Feature/model scale: *d*_model_ > 256 yields diminishing returns; progressive training from 128 → 256 is recommended; *L* > 4 layers provides no significant gain for IDS tasks.

Communication: Parameter upload traffic scales linearly with *N*, but on a 1 Gbps LAN, bandwidth utilization remains <3% even at *N* = 100; for WAN scenarios, enabling 8-bit quantization combined with Top-K (0.1%) sparsification reduces communication volume by an additional 12×.

In summary, TrMulS maintains near-linear scalability within the “sweet spot” of *N* ≤ 20, *M* ≤ 1 M, and *D* ≤ 256. Beyond this region, engineering techniques such as RFF-MMD, dynamic sampling, gradient compression, and asynchronous aggregation keep the time/resource growth slope at ≤1.5×, meeting the scalability requirements of most cross-institutional and cross-domain intrusion detection scenarios.

#### 4.4.6. Discussion on Overfitting Risks of Synthetic Samples and Their Impact on Generalization

TrMulS relies on Exchange-GAN to dynamically generate minority class samples in the cloud, thereby alleviating the extreme class imbalance problem for attack types such as U2R and R2L. However, the “generation-as-learning” paradigm also introduces new overfitting pathways: the generator-discriminator adversarial game, when repeatedly played on small minority class samples in the target domain, tends to memorize noise or local features, causing the synthetic sample distribution to undergo “spurious convergence” with respect to the true distribution.

As indicated in Table 13, a balanced proportion (20–40%) of synthetic samples yields the maximum gain; however, Recall begins to decline when the proportion exceeds 60%, indicating a degradation in generalization capabilities caused by generative overfitting.

1. Mechanism Analysis
  - Fragile small-sample support set: U2R contains only 67 samples; within 200 epochs, the generator can easily memorize all payload byte sequences. Consequently, the Maximum Mean Discrepancy (MMD) minimization no longer reflects the true distribution, but rather a “memorized distribution.”
  - Discriminator provides erroneous signals: The discriminator is likewise trained on an extremely small sample set, causing its decision boundary to also overfit. The gradient signals received by the generator encourage replication of the training set rather than exploration of the data manifold.
  - Gradient leakage to the global model: Federated aggregation propagates the “memorized” generative feature weights to all source domains, causing the global model’s decision boundary for U2R to over-concentrate on 3–4 spurious templates. Any slight variation in the test set leads to missed detections (false negatives).
2. Countermeasures and Improvements

(1) Early Stopping for Generation + Quality Gate

Currently, an MMD < *ε* filter (*ε* = 0.05) is implemented on the server side, but *ε* remains fixed. We propose replacing this with a sliding-window *ε*-schedule: $\varepsilon (t)=\varepsilon_{0}\cdot \exp(-t/\tau )$, where *τ* = 15 rounds. This ensures rapid augmentation in early stages while progressively raising the threshold in later stages, compelling the generator to explore new regions. Experiments demonstrate that the *ε*-schedule improves Recall from 71.2% to 78.9% in the 100% synthetic scenario.

(2) Diversity Regularization

A coverage loss term is added to the generator loss: $L_{\mathrm{cover}}=-\log\left(\det\left(\mathrm{Cov}(f_{G})\right)\right)$, which encourages maximization of the feature covariance matrix determinant, thereby expanding the feature space volume. With *λ*_cover_ = 0.01, the unique 3-g ratio recovers to 0.62, FID decreases to 49, and Recall improves by 4.7%.

(3) Hybrid Weight Decay

A down-weighted cross-entropy loss is applied to synthetic samples: $w_{\mathrm{syn}}=w_{\mathrm{real}}\cdot \gamma$, where γ∈[0.4,0.7] decreases linearly with training rounds. This prevents synthetic samples from dominating the gradient in later stages. With *γ* = 0.5, Recall at 80% synthetic ratio improves from 79.5% to 85.1%.

(4) Adversarial Validation Set

10% of real U2R samples are reserved as a frozen validation set. Generation is halted only when F1 on this set fails to improve for 3 consecutive rounds, thereby avoiding meaningless iterations. This strategy reduces total training time by 22% while improving final F1 by 1.3%.

(5) Out-of-Distribution (OOD) Detection

During target domain deployment, a synthetic sample detector is added: a one-class Siamese Neural Network (SNN) determines whether new samples fall within the “generated template region.” If the score exceeds threshold θ, the confidence is reduced by 0.15 and manual review is triggered, preventing memorized templates from directly influencing decisions.

3. Conclusions and Recommendations

In scenarios with extremely rare classes (fewer than 100 entries), the proportion of synthetic samples should be controlled within the range of 20–40%. When combined with *ε*-scheduling and diversity regularization, this can achieve an 8–10% gain in Recall without sacrificing generalization capabilities.

When the proportion of synthetic data exceeds 60%, spurious distribution convergence emerges even with MMD filtering. To mitigate this, multiple safeguards must be introduced, including down-weighting, early stopping, and Out-of-Distribution (OOD) detection.

Future plans involve introducing Diffusion models (*T* < 50 steps) to replace GANs. This aims to leverage the inherent spatial coverage advantages of the noising-denoising process to further reduce the risk of memorization.

In summary, while synthetic data is undoubtedly a highlight of TrMulS, “generation does not equal gain.” Only under the collective constraints of small sample sizes, quality gating, upper limits on proportions, and diversity regularization can the system enjoy the recall improvements brought by class balancing while avoiding the backlash of synthetic overfitting on generalization ability.

## 5. Conclusions

This paper addresses the limitations of existing intrusion detection methods in terms of cross-domain adaptability, privacy preservation, and minority-class attack detection by proposing an innovative Transformer-based multi-source transfer learning framework, namely TrMulS—a systematic intrusion detection framework that integrates multiple well-established techniques. By effectively coordinating CNN-Transformer, federated learning, MMD alignment, and an improved Exchange-GAN, TrMulS achieves significant improvements in cross-domain adaptability, privacy protection, and class balance. The framework demonstrates synergistic advantages and empirical gains in privacy-sensitive, cross-domain, and highly imbalanced scenarios. Ultimately, TrMulS enables efficient cross-domain knowledge transfer and class-balanced detection under privacy constraints, offering a novel paradigm for the cybersecurity domain that simultaneously addresses detection efficacy, privacy preservation, and class balance. TrMulS’s CNN-Transformer cascaded structure and multi-source feature distribution alignment mechanism (MMD) effectively address cross-domain feature heterogeneity issues. The innovative integration of federated learning architecture and homomorphic encryption technology enables cross-institutional knowledge sharing while protecting data privacy. The improved Exchange-GAN technique fundamentally resolves the scarcity of minority class samples. The experimental results on five intrusion detection datasets demonstrate the effectiveness of the algorithmic framework. Despite the significant achievements of TrMulS, several limitations remain, the current experimental environment is limited to single-machine simulation, which lacks validation under real-world operational constraints. Future research will focus on the following directions: (1) conducting experimental validation in authentic federated learning environments, (2) reducing the computational overhead of the transformer architecture to accommodate resource-constrained IoT edge devices, and (3) integrating meta-learning techniques to enable rapid identification of and adaptation to unknown attack types.

## Figures and Tables

**Table 1:** Average accuracy (Acc), precision (Pre), and recall (%) of the algorithms on the ISCX2012 dataset. The best results are highlighted in bold.

Algorithm	Normal			Infiltrating			HttpDoS			DDoS			BFSSH		
	ACC	Pre	Rec	ACC	Pre	Rec	ACC	Pre	Rec	ACC	Pre	Rec	ACC	Pre	Rec
CNN-Transformer	97.2	96.8	97.5	82.1	79.3	75.4	95.1	94.7	95.3	96.8	96.0	97.1	83.5	80.2	78.6
IDS-INT	96.5	95.1	96.8	84.3	80.1	77.2	94.2	93.5	94.0	95.7	94.8	95.9	85.1	81.0	80.3
ITL-IDS	95.8	94.3	95.2	86.2	82.4	80.1	93.6	92.1	93.8	94.9	93.7	94.5	86.7	82.5	81.7
FETLSVMP	97.8	96.5	97.2	88.5	85.3	83.6	96.0	95.2	95.8	97.2	96.3	96.9	88.2	84.1	83.0
IMDA	98.1	97.2	97.8	89.3	86.0	85.2	96.5	95.8	96.3	97.6	96.8	97.4	89.0	85.3	84.1
**TrMulS**	**98.7**	**98.3**	**98.5**	**93.6**	**91.8**	**90.5**	**97.8**	**97.2**	**97.5**	**98.5**	**98.0**	**98.3**	**94.2**	**92.5**	**91.8**

**Table 2:** Average accuracy (Acc), precision (Pre), and recall (%) of the algorithms on the KDD99 dataset. The best results are highlighted in bold.

Algorithm	DoS			Normal			Probe			R2L			U2R		
	ACC	Pre	Rec	ACC	Pre	Rec	ACC	Pre	Rec	ACC	Pre	Rec	ACC	Pre	Rec
CNN-Transformer	98.5	97.8	98.2	97.3	96.5	97.0	93.1	91.2	90.8	81.2	78.5	75.3	72.5	70.1	68.4
IDS-INT	97.8	96.5	97.2	96.2	95.3	95.8	92.0	90.1	89.5	82.5	79.8	77.1	74.8	72.3	70.5
ITL-IDS	97.2	95.8	96.5	97.7	94.6	95.0	91.3	89.2	88.7	83.8	80.5	79.2	76.3	73.8	72.1
FETLSVMP	98.8	98.0	98.5	97.8	97.0	97.5	94.5	93.1	92.8	86.2	83.7	82.5	80.1	77.5	76.8
IMDA	99.0	98.3	98.8	97.5	97.5	97.9	95.2	93.8	93.5	87.5	85.0	84.3	82.3	79.8	78.5
**TrMulS**	**99.5**	**99.0**	**99.2**	**98.8**	**98.3**	**98.5**	**96.8**	**95.5**	**95.2**	**91.8**	**89.7**	**88.5**	**89.6**	**88.2**	**87.3**

**Table 3:** Average accuracy (Acc), precision (Pre), and recall (%) of the algorithms on the NSL-KDD dataset. The best results are highlighted in bold.

Algorithm	DoS			Normal			Probe			R2L			U2R		
	ACC	Pre	Rec	ACC	Pre	Rec	ACC	Pre	Rec	ACC	Pre	Rec	ACC	Pre	Rec
CNN-Transformer	98.8	98.0	98.5	97.8	97.0	97.5	94.2	92.8	92.5	83.5	80.2	78.8	75.3	72.5	71.2
IDS-INT	98.2	97.3	97.8	97.0	96.2	96.7	93.5	91.8	91.2	84.8	81.5	80.1	77.5	74.8	73.5
ITL-IDS	97.7	96.5	97.0	96.5	95.5	96.0	92.8	90.8	90.3	85.7	82.3	81.5	79.0	76.2	75.0
FETLSVMP	99.2	98.5	99.0	98.3	97.8	98.0	95.5	94.2	94.0	88.3	85.8	84.7	82.8	80.2	79.5
IMDA	99.3	98.8	99.1	98.7	98.2	98.5	96.2	94.8	94.5	89.5	87.0	86.3	84.5	82.0	81.2
**TrMulS**	**99.7**	**99.3**	**99.5**	**99.1**	**98.8**	**99.0**	**97.5**	**96.5**	**96.2**	**93.2**	**91.5**	**90.8**	**91.8**	**90.5**	**89.7**

**Table 4:** Average accuracy (Acc), precision (Pre), and recall (%) of the algorithms on the UNSW-NB15 dataset. The best results are highlighted in bold.

Algorithm	Normal			Generic			Dos			Analysis			Worms		
	ACC	Pre	Rec	ACC	Pre	Rec	ACC	Pre	Rec	ACC	Pre	Rec	ACC	Pre	Rec
CNN-Transformer	97.5	96.8	97.0	93.2	90.5	89.0	95.8	94.8	94.2	88.7	85.5	83.8	82.4	79.0	77.5
IDS-INT	97.0	96.2	96.5	92.6	89.8	88.3	95.3	94.2	93.6	88.0	84.8	83.0	81.5	78.2	76.8
ITL-IDS	96.5	95.5	95.8	91.9	88.5	87.0	94.7	93.5	92.8	87.2	83.8	82.0	80.7	77.5	76.0
FETLSVMP	98.0	97.5	97.7	94.5	92.2	90.8	96.5	95.8	95.5	90.3	87.5	86.0	84.8	81.5	80.2
IMDA	98.2	97.8	98.0	95.0	92.8	91.5	96.8	96.2	95.8	91.0	88.5	87.2	85.5	82.8	81.5
**TrMulS**	**98.7**	**98.3**	**98.5**	**96.8**	**95.0**	**93.8**	**97.8**	**97.3**	**97.0**	**93.5**	**91.2**	**90.0**	**88.2**	**86.0**	**84.5**

**Table 5:** Average accuracy (Acc), precision (Pre), and recall (%) of the algorithms on the CIC-IDS2017 dataset. The best results are highlighted in bold.

Algorithm	Normal			Patator			Dos			Web Attack			Infiltration		
	ACC	Pre	Rec	ACC	Pre	Rec	ACC	Pre	Rec	ACC	Pre	Rec	ACC	Pre	Rec
CNN-Transformer	97.8	97.2	97.5	92.15	89.5	88.0	96.3	95.8	95.5	93.7	91.2	90.0	90.4	87.3	85.8
IDS-INT	97.2	96.5	96.8	91.5	88.8	87.5	95.8	95.2	94.8	93.0	90.5	89.2	89.7	86.5	85.0
ITL-IDS	96.9	96.0	96.3	90.8	87.5	86.0	95.2	94.6	94.2	92.3	89.8	88.5	88.9	85.7	84.2
FETLSVMP	98.3	97.8	98.0	93.5	91.0	89.8	97.0	96.5	96.2	94.8	92.5	91.3	92.1	89.0	87.5
IMDA	98.5	98.0	98.2	94.2	92.0	90.5	97.3	96.8	96.5	95.5	93.2	92.0	93.0	90.8	89.3
**TrMulS**	**99.0**	**98.5**	**98.7**	**99.0**	**98.5**	**98.7**	**98.2**	**97.8**	**97.5**	**97.0**	**95.5**	**94.8**	**95.3**	**93.5**	**92.0**

**Table 6:** F1 scores on the five datasets. The best results are highlighted in bold.

Algorithm	NSL-KDD	KDD99	ISCX2012	UNSW-NB15	CIC-IDS2017
CNN-Transformer	87.2	85.1	86.3	85.8	84.5
IDS-INT	88.5	86.3	87.5	86.6	85.2
ITL-IDS	89.1	87.0	88.2	87.0	85.8
FETLSVMP	91.8	90.6	91.4	90.2	89.0
IMDA	92.7	91.5	92.3	91.0	89.8
**TrMulS**	**95.4**	**94.5**	**95.1**	**94.8**	**93.5**

**Table 7:** Aggregated confusion matrix (rows: actual; columns: predicted; values in %).

Actual\Predicted	Normal	DoS/Generic	Probe/Analysis	U2R	R2L	Infiltration	BFSSH
Normal	98.9	0.8	0.2	0.0	0.1	0.0	0.0
DoS	0.9	98.2	0.7	0.0	0.2	–	–
Probe	1.1	2.0	96.2	0.2	0.5	–	–
U2R	4.5	2.7	5.1	87.3	0.4	–	–
R2L	6.0	3.8	1.2	0.5	88.5	–	–
Infil.	3.2	–	–	–	–	90.5	6.3
BFSSH	2.1	–	–	–	–	5.4	91.8

**Table 8:** Comparison of the performance of the ablation experiments on NSL-KDD (%). (Focusing on minority class attacks).

Variant Model	U2R-Recall	R2L-Recall	Probe-F1	Average Acc
TrMulS w/o MSTL	76.2	82.1	92.3	97.1
TrMulS w/o GAN	75.2 ↓	80.5 ↓	93.8	97.8
TrMulS w/o Fed	83.5	87.2	94.1	98.2
Full TrMulS	89.7	90.8	96.2	99.1

Note: The symbol "↓" indicates a significant decrease in performance compared to the full model (Full TrMulS).

**Table 9:** Performance comparison of ablation experiments on KDD99 (%). (Focus on U2R/R2L attacks).

Variant Model	U2R-Recall	R2L-Recall	Probe-F1	Average Acc
TrMulS w/o MSTL	72.8	79.5	98.2	97.5
TrMulS w/o GAN	70.1 ↓	76.3 ↓	98.8	98.0
TrMulS w/o Fed	80.5	85.2	99.1	98.6
Full TrMulS	87.3	88.5	99.2	99.5

Note: The symbol "↓" indicates a significant decrease in performance compared to the full model (Full TrMulS).

**Table 10:** Performance comparison of ablation experiments on ISCX2012 (%). (Focus on Infiltration/BFSSH attacks).

Variant Model	Infiltration-Rec	BFSSH-Rec	Normal-F1	Average Acc
TrMulS w/o MSTL	82.3	83.5	98.0	95.8
TrMulS w/o GAN	78.6 ↓	80.2 ↓	98.3	96.5
TrMulS w/o Fed	86.2	87.0	98.7	97.8
Full TrMulS	90.5	91.8	98.5	98.7

Note: The symbol "↓" indicates a significant decrease in performance compared to the full model (Full TrMulS).

**Table 11:** Computational Overhead Analysis.

Metric	Centralized CNN-T	TrMulS (Federated, 10 Clients)	Increase
Trainable Parameters	5.83 M	5.83 M × 10 (local) + 5.83 M (global)	10×
Peak GPU Memory	3.1 GB	8.4 GB (central) + 0.9 GB/source (edge)	2.7×
Single-Epoch Training Time	28 min	73 min	2.6×
Encryption/Decryption Time	—	6.2 s (CKKS, 8192 slots)	75% aggregation latency
Uplink Communication/Source	—	22.5 MB (fp32) (11.3 MB if 8-bit quantized)	—
Downlink Communication/Source	—	22.5 MB (same as above)	—
Federation Frequency	—	Aggregate once every 5 local epochs (T = 100 rounds)	—

**Table 12:** Result of the statistical significance analysis.

Dataset	MeanΔ (%)	95% CI (Δ)	*t*-Value	*p*-Value (One-Tailed)	Conclusion
NSL-KDD	+2.7	[+1.9, +3.5]	7.42	1.2 × 10^−5^	Significant
KDD99	+3.0	[+2.2, +3.8]	8.05	4.7 × 10^−6^	Significant
ISCX2012	+2.8	[+2.0, +3.6]	7.68	7.3 × 10^−6^	Significant

**Table 13:** Performance Under Different Synthetic Sample Ratios (NSL-KDD-U2R).

Synthetic Ratio	0%	20%	40%	60%	80%	100%
Recall (%)	68.4	87.3	89.1	84.6	79.5	71.2
Precision (%)	88.0	90.5	89.8	86.1	82.3	74.5
FID (real, gen)	—	42	44	51	63	78

## Data Availability

The data presented in this study are available on request from the corresponding author.

## Appendix A

Multiperspective features are generated as shown in Formula (A1):

(A1) Fi=ReLU(Convi(xinput))

In Formula (A1), Convi represents the i-th convolution kernel operation, $X_{input}\in {\mathbb{R}}^{H\times W}$. Each three-dimensional feature map (height × width × channel, H×W×C) of the convolution output is converted into a one-dimensional vector, retaining structural information:

(A2) $$Flat_{i}=Flatten(F_{i})=\left[\begin{array}{l} F_{i}^{\left(1,1,1\right)}\\ \cdot \\ \cdot \\ \cdot \\ F_{i}^{\left(H,W,C\right)} \end{array}\right]\in {\mathbb{R}}^{\left(H\times W\times C\right)}$$

The flattened vectors of each branch are concatenated to form a multispace feature subset:

(A3) $$X_{multi}=Concat[Flat_{1},Flat_{2},Flat_{3},Flat_{4}]\in {\mathbb{R}}^{4\times (H\times W\times C)}$$

To prevent the convergence of features in each subspace, orthogonal constraints are added:

(A4) $$\min\sum_{i\neq j}\frac{Flat_{i}^{T}Flat_{j}}{\left||Flat_{i}||||Flat_{j}|\right|}$$

Finally, to prevent dimensionality issues, dimensionality reduction can be achieved through fully connected layers:

(A5) Xmulticompressed=ReLU(Wd·Xmulti+bd)

In Formula (A5), $W_{d}$ is a learnable weight matrix, where each row corresponds to an output feature and each column corresponds to an input feature; $b_{d}$ is a learnable bias matrix, with dimensions consistent with the output feature dimensions.

Spatiotemporal association modeling

Capturing long-range dependencies and time series patterns from local features extracted by CNNs is essential for detecting complex attacks such as U2R and R2L attacks. Therefore, on the basis of the transformer, spatiotemporal overposition encoding is proposed to inject temporal information, and multihead self-attention dynamically establishes the correlation between any features to realize spatiotemporal joint reasoning.

The features output by the CNN in Formula (A5) are mapped to the high-dimensional space:

(A6) $$Z_{0}=X_{multi}^{compressed}\cdot W_{e}+b_{e}(b_{e}\in {\mathbb{R}}^{128\times 256})$$

Formula (A6) outputs a dimension of *d_model_* = 256.

Position encoding is performed using sine–cosine coding:

(A7) $$\left\{\begin{array}{l} PE(pos,2i)=\sin(\frac{pos}{1000^{2i/d_{\mathrm{model}}}})\\ PE(pos,2i+1)=\cos(\frac{pos}{1000^{2i/d_{\mathrm{model}}}}) \end{array}\right.$$

In Formula (A7), *p**o**s* is the position of the feature (such as the packet timing index) in the sequence, *i* is the dimension index, and the physical low-frequency component (small *i*) captures long-period patterns (such as continuous scanning attacks), while the high-frequency component (large *i*) detects short-term mutations (such as U2R outbreaks).

The final input is shown in Formula (A8):

(A8) $$E=Z_{0}+PE(pos)\in {\mathbb{R}}^{B\times L\times 128}$$

## Appendix B

This section outlines the design rationale for key hyperparameters. Note that all experiments were conducted on the identical hardware environment described in Section 4.1, with statistical significance assessed using two-tailed *t*-tests (*n* = 10).

1. Theoretical Lower Bound for transformer depth and *d_model_*

According to the Universal Approximation Theorem for transformers (2021):

$\mathrm{FLOPs}\propto L\cdot d_{\mathrm{model}}^{2}\geq 0.9\cdot |X|\cdot d_{\mathrm{model}}$ (X|≈41 IDS feature dimensions)

Solving this inequality yields $d_{\mathrm{model}}\geq 128$ as the minimum threshold to guarantee convergence.

Failure Cases: When $d_{\mathrm{model}}=128$: Macro-F1 on NSL-KDD reached only 92.1% (↓3.3%), with U2R-Recall at 82.5% (↓7.2%). Further increasing depth to *L* = 8 induced overfitting (Training Accuracy: 99.9% → Test Accuracy: 97.3%, Δ = 2.6%); When $d_{\mathrm{model}}=512$: Peak GPU memory consumption reached 11.7 GB, with per-epoch training time increasing by 87%, while F1 improvement was <0.2%, resulting in negative cost-effectiveness; In summary, The configuration of *L* = 4 and $d_{\mathrm{model}}=256$ represents the Pareto-optimal point under the triple constraints of theoretical lower bound, GPU memory ceiling, and diminishing returns threshold.

2. Oversampling Ratio *β* for Exchange-GAN

Theoretical Basis: As noted by Bishop in Pattern Recognition and Machine Learning, the variance of generated samples for the minority class follows ${\mathrm{Var}}_{\mathrm{gen}}\propto 1/(\beta \cdot N_{\mathrm{minor}})$. When β>1.5, ${\mathrm{Var}}_{\mathrm{gen}}$ falls below the true sampling variance, which tends to introduce spurious patterns.

Failure Cases: *β =* 0.8 (undersampling) → U2R-Recall dropped sharply by 14.5% (Table 8, TrMulS w/o GAN); *β =* 1.5 → FID of generated samples increased from 42 to 63, Macro-F1 decreased by 1.1%, and “mode collapse” was observed. Therefore, *β* = 1.2 lies within the theoretically safe interval between “gap filling” and “distribution drift.”

3. MMD Threshold *ε*

The standard error of MMD is given by $\mathrm{SE}(\mathrm{MMD})\approx \sqrt{2n^{-2}\cdot \mathrm{tr}(K^{2})}\approx 0.018$ (with *n =* 2000 and RBF kernel *γ =* 0.5). Applying the 2*σ* criterion yields ε=2×SE≈0.036. In practice, we set *ε* = 0.05 to allow a 25% engineering margin.

Failure Cases: *ε* = 0.02 → 38% of genuine minority class samples were filtered out, causing Recall to drop by 3.7%; *ε* = 0.10 → Low-quality generated samples were admitted, causing Precision to drop by 4.1%. Therefore, *ε* = 0.05 represents a balanced value combining statistical significance with engineering margin.

4. Joint Sensitivity Coupling Experiment

With *L* = 4 and *d_model_* = 256 held fixed, we conducted a *β × ε* two-factor 5×5 grid experiment.

**Table A1:** Joint sensitivity analysis of hyperparameters *β* and *ε.*

ε\β	0.8	1.0	1.2	1.4	1.6
0.02	82.1	84.3	85.9	84.8	82.5
0.05	83.7	87.2	89.7	87.5	85.1
0.10	84.0	86.8	86.4	84.9	82.3

It can be observed that (1.2, 0.05) constitutes the global maximum, with no other extrema within a 1% neighborhood. This confirms that the combination is not a result of “manual trial-and-error luck,” but rather the unique optimal point on the response surface.

The transformer depth and width, *β*, and *ε* have all been determined through a three-level validation process: theoretical lower bounds, failure case analysis, and statistical response surface methodology. This ensures direct reproducibility on other IDS datasets without requiring hyperparameter re-tuning.
